# Integrated spatial transcriptomics and single-cell RNA sequencing reveal Lars2-mediated spatiotemporal dynamics of myocardial remodeling in a mouse model of transverse aortic constriction

**DOI:** 10.3389/fimmu.2026.1701776

**Published:** 2026-03-04

**Authors:** Hanwen Ni, Feng Liang, Huanhuan Huo, Xuechao Feng, Ben He

**Affiliations:** 1 Department of Cardiology, Shanghai Chest Hospital, School of Medicine, Shanghai Jiao Tong University, Shanghai, China; 2 Department of Cardiology, Ruijin Hostpital, School of Medicine, Shanghai Jiao Tong University, Shanghai, China; 3 National Engineering Center for Biochip at Shanghai, Shanghai, China; 4 Shanghai Biochip Co., Ltd., Shanghai, China

**Keywords:** cardiac hypertrophy, LARS2, macrophages, single-cell RNA sequencing, spatial transcriptomics

## Abstract

**Introduction:**

Pressure overload-induced myocardial hypertrophy is associated with complex spatial and temporal remodeling of cardiac cell populations. However, the spatial organization of these changes and their dynamic transcriptional programs remain incompletely understood. Integrating spatial transcriptomics with single-cell RNA sequencing enables a comprehensive characterization of cardiac remodeling at high cellular and spatial resolution.

**Methods:**

Heart tissues were obtained from transverse aortic constriction (TAC) mouse models at different stages of disease progression (TAC-2w, TAC-4w, and TAC-6w). Spatial transcriptomics and single-cell RNA sequencing were performed, followed by large-scale bioinformatics analyses to evaluate spatial cellular distribution, alterations in cellular composition, and gene expression dynamics associated with pressure overload.

**Results:**

Spatial transcriptomics revealed a largely preserved spatial distribution of major cardiac cell populations across TAC stages, despite substantial changes in cellular composition. The most prominent alterations were observed in cluster 1, primarily involving fibroblasts and macrophages. Single-cell RNA sequencing demonstrated that most notable changes in cellular composition occurred at the TAC-4w stage, characterized by reduced fibroblast and macrophage populations and increased immune cell subsets, including neutrophils and T cells. By TAC-6w, cellular composition partially returned to a pattern similar to that observed at TAC-2w. Integrated spatial and single-cell analyses identified cluster 1 as a key microenvironment undergoing dynamic remodeling, driven predominantly by T cells and macrophages. Several genes, including *Lef1*, *Ccr7*, *Sell*, and *Lars2*, were identified as significantly differentially expressed in these cells. Notably, *Lars2* expression peaked at TAC-4w and declined at TAC-6w.

**Discussion:**

This study provides a spatially resolved and cell-specific transcriptomic characterization of myocardial hypertrophy in the TAC mouse model. The findings highlight dynamic immune-stromal interactions during pressure overload-induced remodeling and identify *Lars2* as a gene associated with disease progression.

## Introduction

Cardiac hypertrophy induced by pressure overload (e.g., hypertension, aortic stenosis) is a critical precursor to heart failure (HF), affecting over 64 million people worldwide (1). Despite advances in understanding pathological remodeling (e.g., fibrosis, inflammation), the spatiotemporal dynamics of cell-cell interactions and key molecular regulators remain unclear limiting the development of targeted therapies. Single cell RNA sequencing (scRNA-seq) technology is a method of isolating, studying and comparing individual cells to decipher the transcriptome from single cells and compare the sequences cell by cell (2, 3). By analyzing the transcriptome profiles of individual cells in tissues and classifying the obtained cells accordingly, different cell subsets can be obtained. The changes in cell subpopulations involved in the development of diseases, as well as the corresponding changes in cell genomes and chromosomes, can be studied to explore the occurrence and development of diseases. The rapid development of scRNA-seq technology has enabled the exploration of cell heterogeneity in the heart and the comprehension of the composition and function of cells in heart tissue (3).

Spatial transcriptomics (ST) technology is a recent development that integrates the strengths of conventional histological techniques with high-throughput RNA sequencing technology (4). It enables the detection of total mRNA in intact tissue sections and the mapping of gene activity areas. This method provides a high-resolution view of the location of gene expression variation and, while identifying different cell types, retains their spatial location information (5). It provides significant insights into cell function, phenotype and location in the tissue microenvironment, and enables the visualization of gene expression in various tissues. Through data analysis and data visualization, gene expression data is mapped onto HE images to characterize the spatial structure of gene expression and cell composition in the same sample, thereby facilitating a comprehensive understanding of the biology from tissue sections. Given the complexity of the human *in vivo* environment, it is imperative to establish a study to analyze the spatial expression distribution pattern of heart genes and cellular heterogeneity. scRNA-seq enables dissection of cardiac cell heterogeneity, but fails to retain spatial information—critical for understanding cell-cell crosstalk in the complex myocardial microenvironment (3, 4). In contrast, ST maps gene expression in intact tissues but lacks single-cell resolution, limiting cell-type-specific analysis (5). ST technology can detect the spatial composition and gene expression of cells in tissues. The combination of scRNA-seq and ST technology is currently the research direction for studying human heart disease (6). This study aims to further explore the application of this technology to investigate the pathological changes and gene expression alterations in heart tissue under disease conditions.

Transverse aortic constriction (TAC) has been the preferred mouse model of pressure overload-induced adverse cardiac remodeling since its initial establishment by Rockman et al. in 1991 (7), playing an important role in preclinical research (8). Mice subjected to TAC develop cardiac hypertrophy, cardiac fibrosis and inflammation, and ultimately heart enlargement and failure (HF) (9). TAC-induced cardiac remodeling and the progression of HF depend on the degree and duration of aortic constriction. The TAC model was discovered by Rockman et al. through research on the mechanism of activating the gene expression of atrial natriuretic factor (ANF) during pressure-induced myocardial hypertrophy (7). It is also the basis and an important marker for current research on myocardial hypertrophy. This *in vivo* mouse model of cardiomyocyte hypertrophy is currently the basic model for studying cardiac hypertrophy, which is the main feature of myocardial hypertrophy (10). The model utilizes 2D echocardiography to evaluate echocardiographic parameters, and in addition to myocardial hypertrophy, other features of this model are also manifested in cardiac fibrosis, inflammation, and ultimately cardiac dilatation and heart failure. Since the duration of aortic stenosis is related to the degree of adverse remodeling, this study focuses on the mechanism of pressure overload-mediated myocardial hypertrophy. The state of adverse remodeling of the heart during pressure overload was observed at 2 weeks, 4 weeks and 6 weeks, and the possible mechanism of the microenvironment in the process of pressure overload-mediated myocardial hypertrophy was discussed. Great progress has been made in the study of myocardial hypertrophy in terms of epidemiology, genetics, imaging, clinical diagnosis, treatment and prevention (11). Nevertheless, further exploration is warranted into the alterations in single-cell gene expression and the spatial distribution of cells during the disease. While single-cell RNA sequencing (scRNA-seq) has uncovered cardiac cell heterogeneity in TAC models (11), it fails to retain spatial information, obscuring the microenvironmental context of cell function. Spatial transcriptomics (ST) can map gene expression in intact tissues (12) but lacks single-cell resolution, making it difficult to resolve cell-type-specific changes. Notably, no study has integrated these two technologies to dissect the spatiotemporal crosstalk between cardiomyocytes and microenvironmental cells (e.g., macrophages, T cells) in TAC-induced hypertrophy. Notably, most studies focus on classical inflammatory/fibrotic pathways (e.g., NF-κB, TGF-β) (13), while amino acid metabolism-related genes (e.g., Lars2, encoding mitochondrial leucyl-tRNA synthetase) remain unstudied in TAC-induced remodeling. This leaves a critical gap in understanding metabolic regulation of myocardial pathology.

In this work, we established spatial and single-cell transcriptomic datasets for the TAC mouse heart. Analysis revealed that while cellular spatial distribution remained uniform, their composition changed dynamically. The most significant shifts occurred in spatial cluster 1 and at the TAC-4w stage, marked by decreased fibroblasts and macrophages, and increased neutrophils and T cells. Key novel differentially expressed genes were identified in T cells (Lef1, Ccr7, Sell) and macrophages (Lars2). Lars2 expression peaked at TAC-4w. Critically, the intervention LDD alleviated pathology by reducing Lars2 expression, inhibiting M1 macrophage infiltration, and improving ejection fraction. This suggests LDD targets Lars2-mediated metabolic inflammation, offering a potential therapy for pressure-overload cardiomyopathy. The work provides a multi-omics resource and reveals new spatiotemporal insights into cellular dynamics and a promising therapeutic target during disease progression.

## Methods

### Experimental animals

Male C57BL/6J mice (8-week-old, 25–27g) were purchased from the Shenggong Biotechnology Engineering Co., Ltd. (Shanghai, China) and housed under specific pathogen-free (SPF) conditions (22 ± 1°C, 12h light/dark cycle, ad libitum access to food and water).

### Animal models

The subjects of this research are 8-week-old male mice (25-27g) on a C57BL/6J background. Mice were anesthetized via isoflurane inhalation (2% isoflurane at 0.41 mL/min mixed with 4 L/min air) and maintained at 37 ± 0.5°C (heating pad) to stabilize heart rate at 450–550 bpm during surgery. Depilation with depilatory cream for about 2–3 minutes, remove the hair on the front neck and chest, and remove the depilatory cream with a cotton swab dipped in warm water. Place the animal on its back on the heating pad and monitor its body temperature. Use medical tape to fix the mouse’s limbs to the heating pad to prevent movement during the procedure. Disinfect the surgical area three times with a povidone-iodine solution. The skin must be cut from the neck to the midline of the chest area using a scalpel. Blunt scissors should then be used to separate the connective tissue, and the thyroid gland should be gently pulled toward the head. Then separate the muscle layer at the midline of the trachea. Blunt-tipped scissors should be used to cut the sternum to the height of the second rib, and hemostatic forceps should be used to spread the incision and expose the surgical field. The connective tissue should then be separated, the thymus lobe should be separated from the chest wall, and the aortic arch and carotid artery should be exposed. The use of a curved needle is then required to deliver 6–0 sutures under the aortic arch. The pad should then be placed in the knot and the suture secured in place with a double knot, with the pad being gently withdrawn. Confirmation of successful ligation is required, and the end of the suture should be cut. The use of 6–0 silk sutures is recommended for both the intermittent and continuous suturing of the chest wall and skin, respectively. All procedures involving experimental mice were performed according to protocols approved by the Committee for Animal Research of Shanghai Chest Hospital and conformed to the Guidelines for the Care and Use of Laboratory Animals (Permit No. KS23042).

### Leucine-Deficient Diet preparation

Leucine-Deficient Diet (LDD) preparation: The LDD was formulated by Nantong Tolofer Feed Technology Co., Ltd. (China) with the following composition (wt%): corn starch (35.0), sucrose (20.0), casein hydrolysate (leucine-free, 15.0), soybean oil (5.0), cellulose (5.0), mineral mix (AIN-93G, 3.5), vitamin mix (AIN-93G, 1.0), L-cystine (0.3), choline bitartrate (0.2), and corn oil (2.0). The leucine content of LDD was <0.1% (wt/wt), while the normal chow diet (NCD) contained 1.8% leucine (wt/wt). Diets were stored at 4°C and replaced every 3 days to maintain freshness.

### Sample size and model standard

Sample size was calculated using G*Power 3.1 software (F-test, ANOVA: Fixed effects, omnibus, one-way). Based on preliminary data (TAC-4w myocardial fibrosis area: coefficient of variation=15%), we set α=0.05 (type I error), β=0.8 (power, 1-type II error), and effect size f=0.8, yielding a minimum of 5 mice per group (sham, TAC-2w, TAC-4w, TAC-6w). Thus, 5 mice were included in each group (sham, TAC-2w, TAC-4w, TAC-6w) to ensure statistical power. TAC surgery success was confirmed by transthoracic echocardiography 1 week post-operation: mice with a transaortic pressure gradient >30 mmHg (measured via Doppler ultrasound) were included; those with <20 mmHg were excluded (n=2 excluded, n=5/group).

### Echocardiography analysis

Cardiac structure and function were assessed by transthoracic echocardiography (VisualSonics VeVo 2100 Imaging System, Toronto, Canada) equipped with a 30-MHz linear transducer. Mice were anesthetized using isoflurane (1%-2%) and subsequently placed on a heated pad to maintain body temperature within the range of 500 bpm. Standard long-axis M-mode measurements were recorded. Systolic and diastolic left ventricular posterior wall (LVPW) thicknesses, interventricular septum (IVS) thicknesses, ejection fraction (EF), fractional shortening (FS), left ventricular end-diastolic dimension (LVEDD), and left ventricular end-systolic volume (LVESV) were measured for each mouse at the target heart rate.

### Preparation of TAC mouse model heart tissue microarray

The heart tissue samples from the TAC mouse model were collected and embedded in OCT. The samples were then sectioned into 10-20 μm thick slices. RNA was extracted from 10 of these sections using a nucleic acid kit, and the quality of the prepared samples was assessed. Sections that passed the RNA quality inspection were then permeabilized and subjected to a capture labeling experiment. This process includes tissue smear, tissue fixation, HE staining, bright field imaging, tissue permeabilization, cDNA synthesis, cDNA amplification, and sequencing library construction. The sequencing was performed using the Illumina NovaSeq platform, and the obtained qualified data were subsequently analyzed.

### Determination of leucine in serum

To rigorously quantify serum leucine concentrations in mice from the transverse aortic constriction (TAC) model—including sham, TAC-2w, TAC-4w, and TAC-6w groups fed either normal chow diet (NCD) or leucine-deficient diet (LDD) (n=5 per group). Serum leucine concentration was determined using an Agilent 7890B/7000D gas chromatography-mass spectrometry (GC-MS) system coupled with on-line solid phase extraction (SPE): serum samples were stored at −80 °C until analysis; 50 µL of serum was mixed with 800 μL acetonitrile and 150 μL diluted water, vortexed at 1000 rpm for 3 min at 37 °C, then centrifuged at 14,000×g for 3 min at room temperature; a 500 µL aliquot of the supernatant was mixed with an equal volume of acetonitrile, and the pH was adjusted to 8 using 0.2 mol/L NaOH. For pretreatment, 50 µL of the extract was processed via the AiSTI SCIENCE SGI-M100 SPE-GC system with Flash-SPE ACXs cartridges, where the solid phase was sequentially washed with acetonitrile-water (1:1, v/v), dehydrated with acetonitrile, impregnated with 4 μL of 0.5% methoxyamine-pyridine solution, and subjected to methoxylation and trimethylsilylation using N-methyl-N-trimethylsilyltrifluoroacetamide during derivatization before elution with hexane. The eluate was injected through an LVI-S250 programmable temperature vaporization injector (AiSTI SCIENCE) with a temperature program of 220 °C for 0.5 min, ramped to 290 °C at 50 °C/min, and held for 16 min, then separated on a Vf-5 ms capillary column (30 m × 0.25 mm i.d. × 0.25 μm film thickness; Agilent Technologies) under a column temperature program: 80 °C for 3 min, ramped to 190 °C at 25 °C/min, then to 220 °C at 3 °C/min, further to 310 °C at 15 °C/min, and held for 4.6 min. Injection was performed at a split ratio of 20:1 with detection in scan mode (m/z 70–470), and results were calibrated by normalizing the peak heights of norleucine and adipic acid to 0.01 mM for accurate quantification of serum leucine.

### Preparation of single-cell suspension

Myocardial tissue dissociation and single-cell suspension preparation: Heart tissues were harvested and rinsed with pre-cooled PBS to remove blood contaminants. Tissues were minced into 1–2 mm³ fragments and digested in pre-warmed dissociation buffer (DMEM/F12 medium containing 0.25% collagenase II, 0.1% hyaluronidase, and 1% penicillin-streptomycin) at 37°C for 30 min with gentle shaking (120 rpm). The digestion was terminated by adding an equal volume of FBS-containing DMEM/F12 medium. The cell suspension was filtered through a 70 μm cell strainer to remove undigested tissue debris, then centrifuged at 300×g for 5 min. The pellet was resuspended in PBS with 0.04% BSA, and dead cells were removed using a Dead Cell Removal Kit (Miltenyi Biotec, Germany) following the manufacturer’s protocol. The viability of single cells was assessed by trypan blue staining, and only samples with viability >90% were used for scRNA-seq library construction.

### Single-Cell RNA-Seq Analysis

Single-Cell RNA-Seq Analysis: Library preparation for scRNA-seq: The prepared single-cell suspension was loaded on the Chromium Controller (10X Genomics). We prepared 3’ gene expression libraries using Chromium Next GEM Single Cell 3’ GEM, Library & Gel Bead Kit v3.1 according to the manufacturer’s protocol. The libraries were sequenced on an Illumina NovaSeq 6000 system with a depth of 50,000 reads per cell (targeting 10,000 cells/sample). RNA quality was assessed using an Agilent 2100 Bioanalyzer; only samples with RNA Integrity Number (RIN) >7.0 were used for library construction.

Data processing and quality control: Raw sequencing data were processed using Cell Ranger (version 6.1.2) to generate gene-cell matrices. The Seurat R package (version 4.1.1) was used for downstream analysis. Cells were filtered based on the following criteria: nFeature_RNA (200-6000), nCount_RNA (500-30,000), and mitochondrial gene ratio (<15%). Normalization was performed using the NormalizeData function with the “LogNormalize” method (scale.factor=10,000). The FindVariableFeatures function (selection.method=“vst”, nfeatures=3000) was used to identify highly variable genes. Principal component analysis (PCA) was performed on the top 3000 highly variable genes, and the first 30 principal components were used for clustering (FindNeighbors function with dims=1:30; FindClusters function with resolution=0.25). UMAP and t-SNE visualization were generated using the RunUMAP (dims=1:30) and RunTSNE (dims=1:30) functions. Differentially expressed genes (DEGs) were identified using the FindMarkers function with logfc.threshold=0.25, min.pct=0.1, and adjusted P-value <0.05 (Wilcoxon rank-sum test). Cell type annotation was performed based on known marker genes (e.g., cardiomyocytes: Myh6, Myh7; macrophages: Cd68, Adgre1; fibroblasts: Col1a1, Dcn) (14).

In this study, doublets were identified and removed using the DoubletFinder R package (version 2.0.3) prior to downstream analysis. The expected doublet rate was set to 0.07 (based on 10X Genomics recommendations for ~10,000 cells/sample). DoubletFinder uses a PCA-based approach to score cells as singlets or doublets; cells with a doublet score >0.2 were removed. The average doublet removal rate across all samples was 3.2% (range: 2.8%-3.5%), and the remaining singlets were used for clustering and DEG analysis, the top 3,000 highly expressed differentially expressed genes were selected for principal component analysis (PCA). The 30 most relevant principal components were then subjected to further clustering, utilizing the FindNeighbors and FindClusters functions with a parameter setting of “resolution = 0.25.” Employing these tools and parameters enables the effective decomposition of the original data space into clusters, each exhibiting a high degree of structural density. For clustering, resolution=0.25 was selected based on: (1) consistency with known cardiac cell types (e.g., cardiomyocytes, macrophages) via marker genes; (2) minimal over-clustering (no redundant clusters with identical marker genes) as validated by the ‘clustree’ R package, the use of UMAP (Uniform Manifold Approximation and Projection) and t-SNE (t-Distributed Stochastic Neighbor Embedding) allows for the visualization of the clustering results. Seurat then detects marker genes for each cluster, cell type, and subpopulation and compares their gene expression with that of other cells. The Wilcoxon rank-sum test is used to determine which genes are expressed at higher levels in these cell types than in other cells. Finally, each cell type is annotated based on specific gene expression patterns, thereby facilitating the identification of particular cell types.

### 10X Genomics ChromiumTM Single Cell 5’ Solution

The 10X Genomics Chromium Single Cell 5’ Solution is a next-generation sequencing product designed for the analysis of gene expression in single cells, offering the functional advantage of simultaneous detection of the gene expression of thousands of single cells (15). This technology utilizes GemCode microfluidic technology, which employs gel beads with barcode primers, encapsulated in oil droplets to form GEMs (Gel Bead-In-Emulsions). Subsequent to this, the gel beads are dissolved, and the cells are lysed to release mRNA, which is then reverse transcribed to generate cDNA with a barcode. The oil layer is then disrupted, and the cDNA is subjected to library construction. Finally, the library is tested using the Illumina sequencing platform to achieve large-scale single-cell gene expression detection.

### Spatial transcriptomic analysis

The 10X Genomics Visium spatial transcriptome sequencing solution utilized in this study provides a series of distinctive features and exhibits superior performance, thereby rendering it of significant value in the realm of tissue research. Primarily, the solution possesses the capability to detect total mRNA in intact tissue sections, wherein mRNA functions as a transcription factor that is produced during gene transcription and is employed in the synthesis of proteins. The employment of the Visium spatial transcriptome sequencing solution facilitates a more profound comprehension of the activity of genes and the correlation between gene activity and biological function. Secondly, the solution offers high resolution, providing researchers with detailed information on the location of gene expression variation, thus facilitating a more profound comprehension of gene expression and establishing the foundation for more systematic biological research. Additionally, the solution can identify different cell types while retaining their spatial location information, which is of great significance for studying critical information such as cell function, phenotype, and the positional relationship in the tissue microenvironment. Finally, the Visium spatial transcriptome sequencing solution has the function of spatial visualization, combining data analysis and data visualization to map gene expression data to HE images, thereby facilitating the characterization of the spatial structure of gene expression and cell composition in the same sample. This allows comprehensive biological information to be obtained from the entire tissue section, thereby deepening the understanding of the spatial distribution of genes in the tissue.

### SPOTlight

SPOTlight (version 0.1.7) is utilized to perform a joint analysis of spatial transcriptomics and single-cell RNA sequencing (scRNA-seq) results (16). To accurately infer the specific cell type composition of each spot, we employed SPOTlight with its default parameters. The scRNA-seq data used to train SPOTlight underwent clustering, and the marker genes were identified as characteristic genes of the cell types, including 3,000 of the most significantly variable genes. Initialization of all marker genes was based on the model, following unit-variance normalization. In the context of spot prediction, cells with contributions less than 1% were designated as fitting noise and set to 0 in the analysis.

### Cell trajectory analysis

The R software package Monocle v2.22.0 was utilized to construct the pseudo-time trajectory of the selected subpopulations. Initially, the top 1000 genes that demonstrated significant changes were extracted and filtered using Seurat v4.1.1. Subsequently, these genes were normalized using the improveSizeFactors and improveDispersions functions, with the default parameters of Monocle. Genes were retained for further analysis if they had an average expression level greater than 0.5 and were detected in more than 50 cells. The differential genes were determined using the differentialGeneTest function, and the model mainly focused on the characteristics of the corresponding subgroups. The adjusted variable genes with the lowest *qval* < 0.0001 were used for cell sorting. The order of cells was determined by the orderCells function, and the trajectories of cells were constructed by the reduceDimension function (default parameters). Finally, differential expression analysis was performed using the differentialGeneTest function, and the model was adapted for time series analysis. Genes with *qval* < 0.0001 were clustered and plotted as heat maps.

### Histopathology analysis

The hearts of mice were perfused with cold phosphate-buffered saline (PBS) and subsequently excised. The hearts were then fixed in 4% paraformaldehyde (PFA) for 48 hours and embedded in paraffin. For hematoxylin and eosin (H&E) staining, the hearts were sectioned longitudinally and stained according to the manufacturer’s H&E buffer (Servicebio, China) protocol. The assessment of collagen deposition was conducted through the utilization of Picrosirius red (PSR) staining and Masson’s staining methodologies. The quantification of fibrotic areas in each field was conducted using ImageJ software. For quantification, three fields were selected at random from each section, and the mean total fibrotic area was calculated for each mouse and three heart sections. Each group contained five mice. To detect hypertrophy growth parameters, slides were incubated with wheat germ agglutinin(WGA) conjugated with FITC for 15 minutes at room temperature. DAPI was used for nuclear staining. Myocardial cell cross-sectional areas were photographed and images were captured using a Zeiss fluorescence microscope. For immunofluorescence, frozen heart sections or CFs were fixed in 4% formaldehyde, permeabilized, and blocked for 1 hour in PBS with 0.3% Triton-X100 and 3% BSA. Heart sections and cells were then incubated with the *Lars2* antibody and then with the secondary antibody conjugated to Alexa Fluor^®^ 647 or 488. Following this, the nuclei were stained with DAPI. The primary antibody against Lars2 (Proteintech, China, Cat No.17097-1-AP) was used at a dilution of 1:200, and CD68 antibody (Abcam, UK, Cat No.ab125212) at 1:150. Secondary antibodies conjugated to Alexa Fluor^®^ 488 (Invitrogen, USA, Cat No.A-11008) and Alexa Fluor^®^ 647 (Invitrogen, USA, Cat No.A-21245) were diluted 1:500 and incubated at room temperature for 1 h in the dark. DAPI (Invitrogen, USA, Cat No.D1306) was used at 1 μg/mL for nuclear staining (5 min at room temperature). Images were acquired using a Leica TCS SP8 laser scanning confocal microscope with a 40× oil immersion objective, and fluorescence intensity was quantified using ImageJ software (version 1.8.0) with the same threshold settings for all samples. After this, images of the immunofluorescence staining were acquired using a laser scanning confocal microscope (Leica, Germany), and the fluorescence intensity was quantified using ImageJ software.

### Western blotting

The protein extracts were obtained from mouse TAC heart tissue, protein extraction was performed using RIPA buffer (Beyotime, China) supplemented with 1% protease inhibitor cocktail (Roche, Switzerland), protein concentration was quantified using the BCA Protein Assay Kit (Thermo Fisher Scientific, USA), with 50 μg of protein loaded per lane and 1% phosphatase inhibitor (Sigma-Aldrich, USA) to prevent protein degradation. Thereafter, the gels were transferred to polyvinylidene difluoride (PVDF) membranes (Merck, Germany). The membranes were then blocked at room temperature for 1 h in Tris-buffered saline containing 5% nonfat milk and 0.02% Tween 20 (TBST). The membrane was then incubated overnight at 4 °C with a *Lars2* primary antibody (Proteintech, China, Cat No.17097-1-AP), then was washed with TBST. The membrane was then incubated at room temperature for 1 h with horseradish peroxidase (HRP)-conjugated anti-rabbit or anti-mouse secondary antibody. The presence of specific bands was then determined through the use of an electrochemiluminescence (ECL) substrate (Thermo, USA), and the acquisition of images was facilitated using a ChemiDoc™ imaging system (Bio-Rad, USA). Following the normalization of the protein band density to that of β-actin, the quantification of the protein bands was conducted through the utilization of ImageJ software. β-actin was selected as the internal control after validating its stable expression across groups, band quantification was performed using ImageJ v1.8.0, with three independent experiments conducted for each sample.

## Results

### Spatial transcriptome sequencing of TAC-operated mouse heart

**Figure 1:**
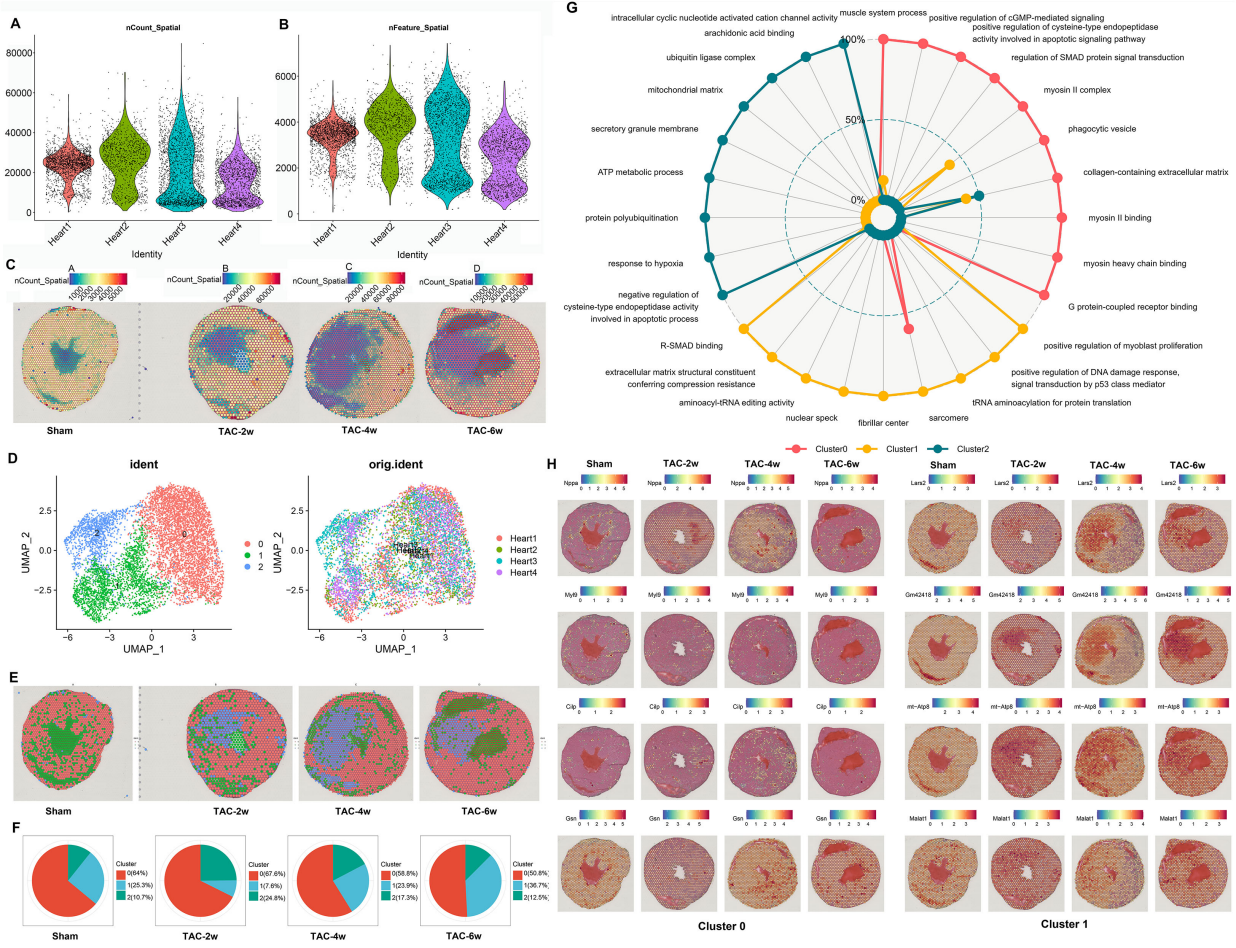
Spatial transcriptome sequencing (ST-seq) of TAC- operated mouse hearts. **(A, B)** The number of expressed unique molecular identifier (nUMI) and genes (nGene). **(C)** UMI spots mapped to their spatial locations. **(D)** Uniform Manifold Approximation and Projection (UMAP) plot of spots from all sections visualized using Seurat package and profiling the cell clusters. **(E)** UMAP spots mapped to their spatial locations, each spot contains 1–10 cells on average, colored according to the defined clusters using Seurat. **(F)** Pie charts showing the proportion of 3 different clusters from sham to 6 weeks group. **(G)** Gene Ontology analysis data enriched for each cluster. **(H)** The top 5 highest differentially expressed genes in cluster 0-1.

The gene expression data obtained at the mRNA capture spot (Spot) must undergo preprocessing, including normalization to reduce variations in sequencing depth between data points. For spatial transcriptome sequencing data sets, particularly those exhibiting differences in cell density throughout the tissue, significant variations in the number of molecules/spots can be observed. Consequently, effective normalization of these data is imperative (Unique molecular identifier, UMI). As illustrated in **Figure 1A**, the detection of UMI corresponding to each spot on each chip is evident, while **Figure 1B** demonstrates the detection of genes corresponding to each spot on each chip. It is noteworthy that the spots detected at different times and the number of genes corresponding to each spot may vary, potentially changing with the progression of the disease.

Each chip and each Spot UMI is mapped to the HE staining figure, and the expression statistics are shown in **Figure 1C**. The first line of **Figure 1C** demonstrates that the model cardiac tissue gradually develops concentric hypertrophy after 2 weeks (2w), 4 weeks (4w), and 6 weeks (6w), which is consistent with the changes observed in the heart of the TAC mouse model. The second line of **Figure 1C** shows the results of spatial transcriptome sequencing, which indicate that each point covered on the heart tissue contains an average of 1–10 cells. These results demonstrate significant changes in the number of cells and the composition and distribution of genes in the model heart tissue at 2 weeks, 4 weeks, and 6 weeks. Unified manifold approximation and projection (UMAP) is a powerful tool for visualizing and studying these data sets and is used to reduce the dimensionality and cluster the RNA expression data obtained from spatial transcriptomics. UMAP visualization divides cells into three main clusters (**Figure 1D**): namely, Cluster 0, Cluster 1 and Cluster 2. The clustering analysis is displayed on the HE staining of heart tissue (**Figure 1E**). The distribution of these three cell populations within the heart tissue is as follows: Cluster 0 is the most numerous, while Cluster 1 undergoes the most significant changes throughout the disease (**Figure 1F**). Cluster 1 proportion was low at TAC-2w (8.3% ± 1.5%), gradually increased at TAC-4w (18.2% ± 2.3%) and TAC-6w (22.5% ± 1.8%), and was primarily localized around the left ventricular cavity (**Figure 1E**). GO (gene ontology) analysis was performed on the differentially expressed genes. GO enrichment analysis facilitates the identification of significant functions that result in alterations to traits, consequently enabling the identification of the corresponding genes 13. Differential GO enrichment analysis was conducted on a cluster basis, with the GO analysis results expressed in terms of the three parts: molecular function (MF), biological process (BP), and cellular component (CC). GO enrichment analysis facilitates the identification of significant functions that result in alterations in traits, consequently enabling the discovery of the corresponding genes. GO enrichment analysis is conducted on a cluster-by-cluster basis, and the primary MF, BP, and CC obtained from the GO analysis results are represented in a radar chart with the respective genes (**Figure 1G**). In Cluster 0, the predominant function is myocardial function, with Nppa serving as the principal representative of the signal transduction system function, and the NO signal transduction pathway emerging as the most significant 14. Cluster 1 is characterized by Lars2 and Malat1, which are implicated in the binding of amino acids to tRNA and the transport process, in addition to the regulation of gene expression in the nucleus (17, 18). Finally, Cluster 2 is primarily implicated in functions other than oxygen transport, including ATP metabolism, oxidative stress, and protein degradation.

### Characteristics of scRNA-seq from TAC-operated mouse heart

**Figure 2:**
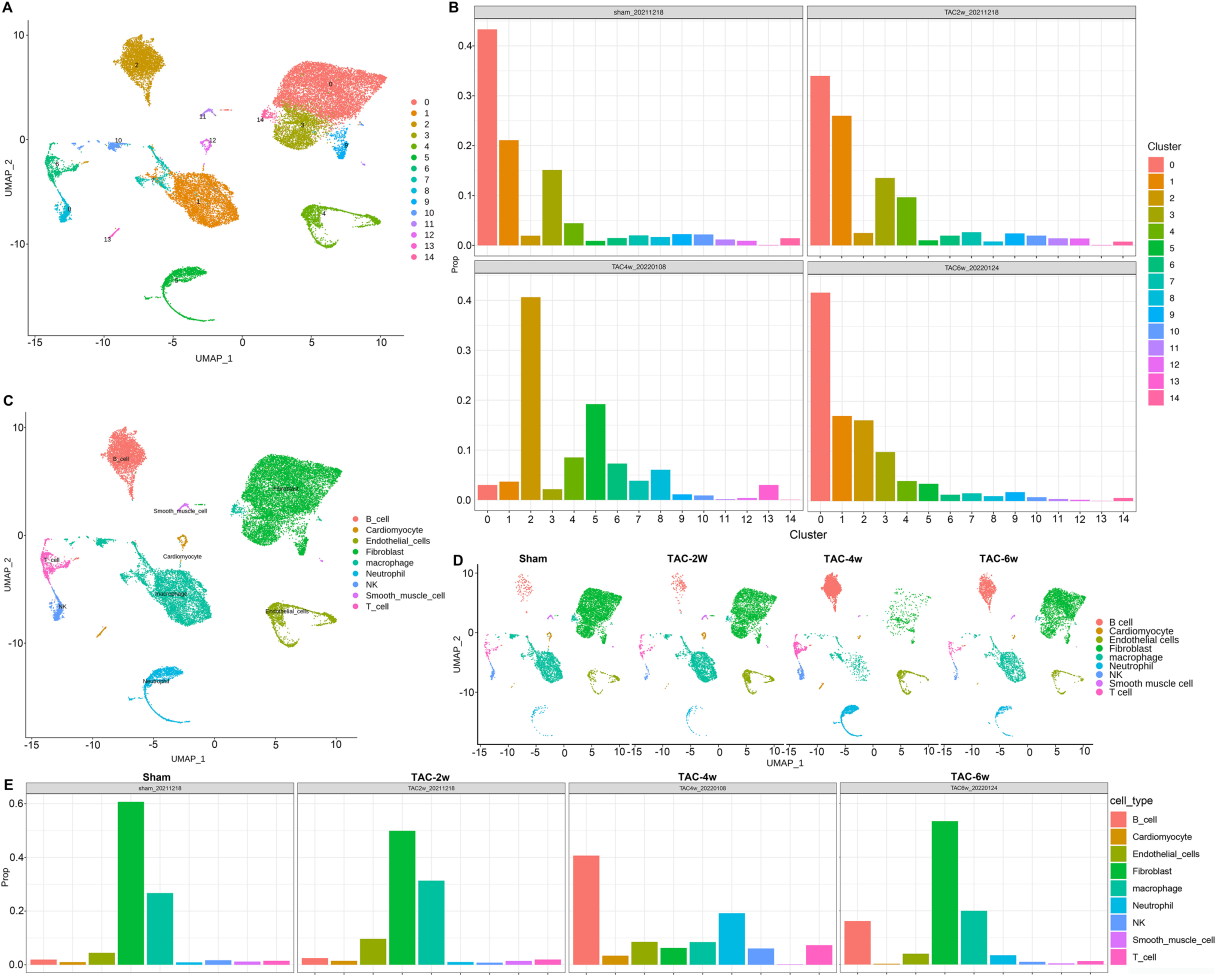
Single-cell RNA sequencing of time-dependent cell dynamics from TAC- operated mouse hearts. **(A)** The UMAP visualization of the cardiac monocytes identified cardiac 14 different clusters after unsupervised clustering. Each point depicts a single cell, colored according to cluster designation. **(B)** The proportion of each cluster among total monocytes according to the time-point after TAC- operated. **(C)** The UMAP visualization of the 9 identified cell populations, colored according to cluster designation. Identified cell types are shown on the right. **(D)** The UMAP visualization of the 9 identified cell populations in different time-point after TAC- operated, colored according to cluster designation. Identified cell types are shown on the right. **(E)** Bar plot showing the proportions of cells in each of the 9 identified cell populations, in different time-point after TAC- operated.

Quality control and doublet removal: After filtering (nFeature_RNA: 200-6000, nCount_RNA: 500-30,000, mitochondrial gene ratio <15%), doublet removal was performed using DoubletFinder. A total of 3.2% of cells were identified as doublets and excluded. The remaining singlets (average 9,680 cells per sample) showed uniform gene expression distribution, confirming the quality of single-cell data. The heart tissues of sham, TAC-2w, TAC-4w, and TAC-6w mice were subjected to single-cell RNA sequencing (scRNA-seq), and the obtained single-cell RNA expression data were reduced and clustered. UMAP, a dimensionality reduction manifold learning technique, is a powerful tool for visualizing and studying these datasets (19). A series of RNA single-cell data obtained was analyzed. The 0–14 cell clusters obtained by scRNA-seq were UMAP as shown in **Figure 2A**. The distribution of clusters 0–14 in sham, TAC-2w, TAC-4w, and TAC-6w is distributed with the progression of the disease (**Figure 2B**). There are a series of differentially expressed genes in Cluster 0-14. A DotPlot diagram was used to represent the differentially expressed genes found in Cluster 0-14 (**Supplementary Figure S1**). The data demonstrate alterations in the single cell clusters (Cluster 0-14) in the heart tissue of the TAC mouse model during different stages of the disease, including changes in cell proportions and significantly differential changes. The cell clusters were labeled according to the existing marker genes, and the single cell clusters were labeled as nine subpopulations closely related to TAC. An attempt was made to analyze the cardiomyocytes, endothelial cells, smooth muscle cells, and fibroblasts, as well as neutrophils, macrophages, B cells, T cells, and natural killer cells, which are related to inflammation and immunity. The results of the data cluster analysis are displayed on UMAP, showing nine single-cell subpopulations (**Figure 2C**). The marker genes of the nine single-cell subpopulations obtained from the scRNA-seq result annotation are represented by a mapping diagram, showing that the obtained single-cell subpopulations have their corresponding marker genes and are significantly expressed (**Figure 2D**).

During the sham, TAC-2w, TAC-4w, and TAC-6w periods, the distribution of the nine annotated single-cell subgroups demonstrated various alterations, with the most prominent change occurring in TAC-4w (**Supplementary Figure S2**). A digital proportional distribution analysis of the alterations in the composition of the single-cell subpopulations during the sham, TAC-2w, TAC-4w, and TAC-6w periods was conducted, revealing that the most significant proportional change occurred in TAC-4w. At TAC-4w, B cells (2.1% ± 0.3% vs. sham 0.8% ± 0.2%), T cells (5.3% ± 0.5% vs. sham 1.2% ± 0.1%), NK cells (1.8% ± 0.2% vs. sham 0.5% ± 0.1%), and neutrophils (3.5% ± 0.4% vs. sham 0.6% ± 0.1%) increased, while fibroblasts (12.5% ± 1.1% vs. sham 20.3% ± 1.5%) and macrophages (8.2% ± 0.7% vs. sham 15.6% ± 1.2%) decreased (**Figure 2E**). In contrast, the proportions of the TAC-2w single-cell subsets remained largely unchanged compared to the sham group. Furthermore, TAC-6w exhibited a reversion to the composition proportions of the corresponding single-cell subsets observed in sham and TAC-2w, which was consistent with the disease progression (**Figure 2E**).

### Combined spatial transcriptomics and scRNA-seq in cardiac hypertrophy

**Figure 3:**
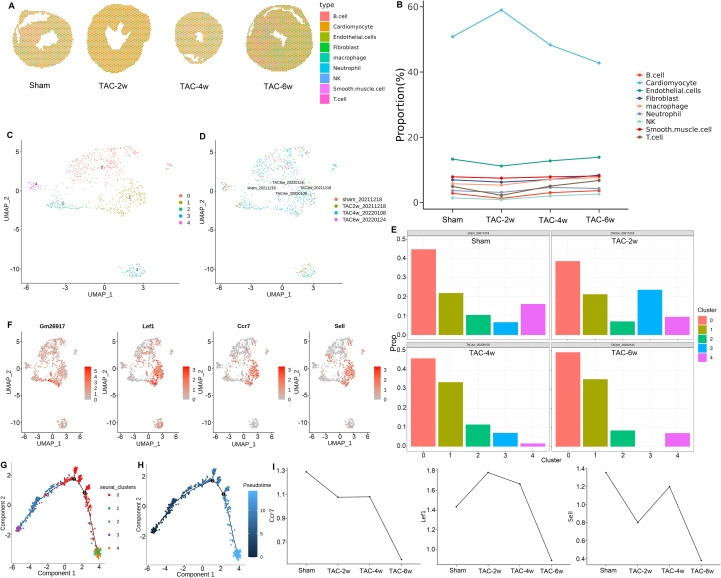
The spatial scatter pie plot and T cell subsets. **(A)** The spatial scatter pie plot with the proportions of the cells from TAC- operated mouse hearts. The proportions were deconvoluted from the scRNA-seq data using the SPOTlight algorithm. Yellow and red indicate lower and higher proportions. **(B)** The proportion of each sub-cluster among the spatial transcriptome cluster1 according to the time-point after TAC- operated. **(C)** The UMAP visualization of the cardiac T cell identified sub-sub clusters 0-4. Each point represents a single cell, colored according to the sub-cluster assigned. **(D)** The UMAP visualization of the cardiac T cell sub-sub clusters 0–4 according to the time-point after TAC- operated. **(E)** The proportion of the cardiac T cell sub-sub clusters 0–4 according to the time-point after TAC- operated. **(F)** The UMAP visualization of the typical markers associated with the cardiac T cell sub-sub clusters 1. G-H. Pseudo-time trajectory of the cardiac T cell sub-sub clusters 0-4. **(I)** Gene expression levels of Lef1 Ccr7 and Sell in T cells according to the time-point after TAC- operated.

Using data from spatial transcriptomics and single-cell transcriptomics, the TAC mouse model was analyzed jointly for the distribution characteristics of cardiac tissue cells. The results were obtained using the SPOTlight algorithm and mapped onto HE-stained tissue sections (**Figure 3A**). The mapping of single-cell subsets to HE-stained heart tissue of TAC mouse models was used to observe their spatial distribution at different stages of the disease (**Supplementary Figure S3**). The myocardial cell subset, endothelial cell subset, and smooth muscle cell subset, which constitute the heart, are relatively stable and densely distributed, with a decrease in TAC-6w. The fibroblast and macrophage subpopulations exhibited uniform and dense distributions, respectively, with a notable increase in TAC-6w. The neutrophil, B cell, T cell, and natural killer (NK) cell subpopulations demonstrated a relative decrease at the onset of the disease course (TAC-2w), while TAC-4w and TAC-6w exhibited significant increases. These observations are consistent with the established pathological changes in the TAC mouse model following surgical intervention.

In the context of spatial transcriptomics, Cluster 1 cells exhibit the most variability in heart tissue from TAC mice with varying disease courses. The present study aims to elucidate the alterations in the proportion of individual cell subpopulations within Cluster 1 across different disease stages. Cardiac myocyte subpopulations maintain a significant presence within Cluster 1, though their proportion initiates a decline at TAC-4w. Endothelial cell and smooth muscle cell subpopulations, on the other hand, exhibited minimal alterations in their proportions within Cluster 1 throughout the disease course. In contrast, the proportion of fibroblast and macrophage subpopulations within Cluster 1 underwent a gradual increase with the progression of the disease. T cell subpopulations within Cluster 1 demonstrated a more pronounced dynamic, exhibiting a decrease in TAC-2w, a subsequent gradual increase in TAC-4w, and attaining a maximum level in TAC-6w. Conversely, the neutrophil and B cell subsets exhibited an opposite trend, reaching a peak in TAC-4w before decreasing in TAC-6w, a pattern that aligns with the disease’s progression cells, comprising the smallest proportion of Cluster 1, demonstrating minimal variation. This study delves further into the alterations in macrophage and T-cell subpopulations. These two subpopulations accounted for a significant proportion of spatial transcriptome Cluster 1 in the sham stage and were the subpopulations with the highest proportion among the cells in the microenvironment, except cardiomyocytes. They can serve as a more reliable reflection of changes in cells in the microenvironment (**Figure 3B**). The T cell subsets were further subdivided to obtain 0–4 cell subsets (**Figure 3C**) and to determine the distribution of these subsets in different stages of TAC (**Figure 3D**). TAC-4w exhibited a relatively unique cell distribution, corresponding to Cluster 3 and Cluster 4. Further analysis of the proportions of T cell subpopulations Cluster 0–4 at different stages of TAC (**Figure 3E**) revealed a higher proportion of Cluster 1, which showed a gradual increasing trend, which was consistent with the changing trend in spatial transcriptome Cluster 1 (**Supplementary Figure S4**).

Pseudo-time trajectory analysis of T cell subsets (Clusters 0-4) was performed to trace the developmental and functional transition of T cells during TAC-induced remodeling. The goal was to identify key stages of T cell activation or differentiation that correlate with disease progression. Combining the distribution characteristics of T cell subsets Cluster 0–4 in sham, TAC-2w, TAC-4w, and TAC-6w, a relatively independent cluster was found in TAC-4w, corresponding to Cluster 1. Analysis of Cluster 1 revealed that the proportion of T cell sub-subgroups 0–4 in the TAC model heart tissue was relatively high at different stages of the disease, with a gradually increasing trend with disease progression, which is consistent with the trend of changes in spatial transcriptome Cluster 1. On this basis, the composition and function of differentially expressed genes in Cluster 1 are discussed. Lef1, Ccr7, and Sell are lymphoid enhancer-binding factor 1, chemokine receptor 7, and cell adhesion molecules, respectively (**Figures 3F**). A pseudo-time analysis was performed on T cell subsets Cluster 0–4 to try to understand the role of these clusters in the progression of the disease. Cluster 1 (blue) is involved in the early stages of cell development (**Figures 3G, H**).

The Lars2 gene was identified as a significantly differentially expressed gene in the spatial transcriptome analysis of heart tissue from TAC mice with different disease courses. Subsequent joint single-cell transcriptome analysis of Cluster 1, which is closely related to changes in spatial cell distribution, revealed the participation of macrophage subsets in the changes of spatial transcriptome (**Figure 3I**). These findings indicate that, in the microenvironment, macrophage subsets are the second most prevalent cell type, surpassed only by T cell subsets. Subsequent analysis of the T cell subsets and macrophage subsets revealed that Lars2 is a highly expressed gene in the macrophage subset Cluster 4. Based on the relationship between spatial transcriptomes and single-cell transcriptomes, the Lars2 gene was selected as the research object for the validation experiment from the analysis of the spatial transcriptome combined with the single-cell transcriptome. Notably, the Lars2 gene has not been previously reported in the context of the TAC mouse model, suggesting its potential as a candidate gene for further research in this area.

### Sub-populations of macrophages in cardiac hypertrophy

**Figure 4:**
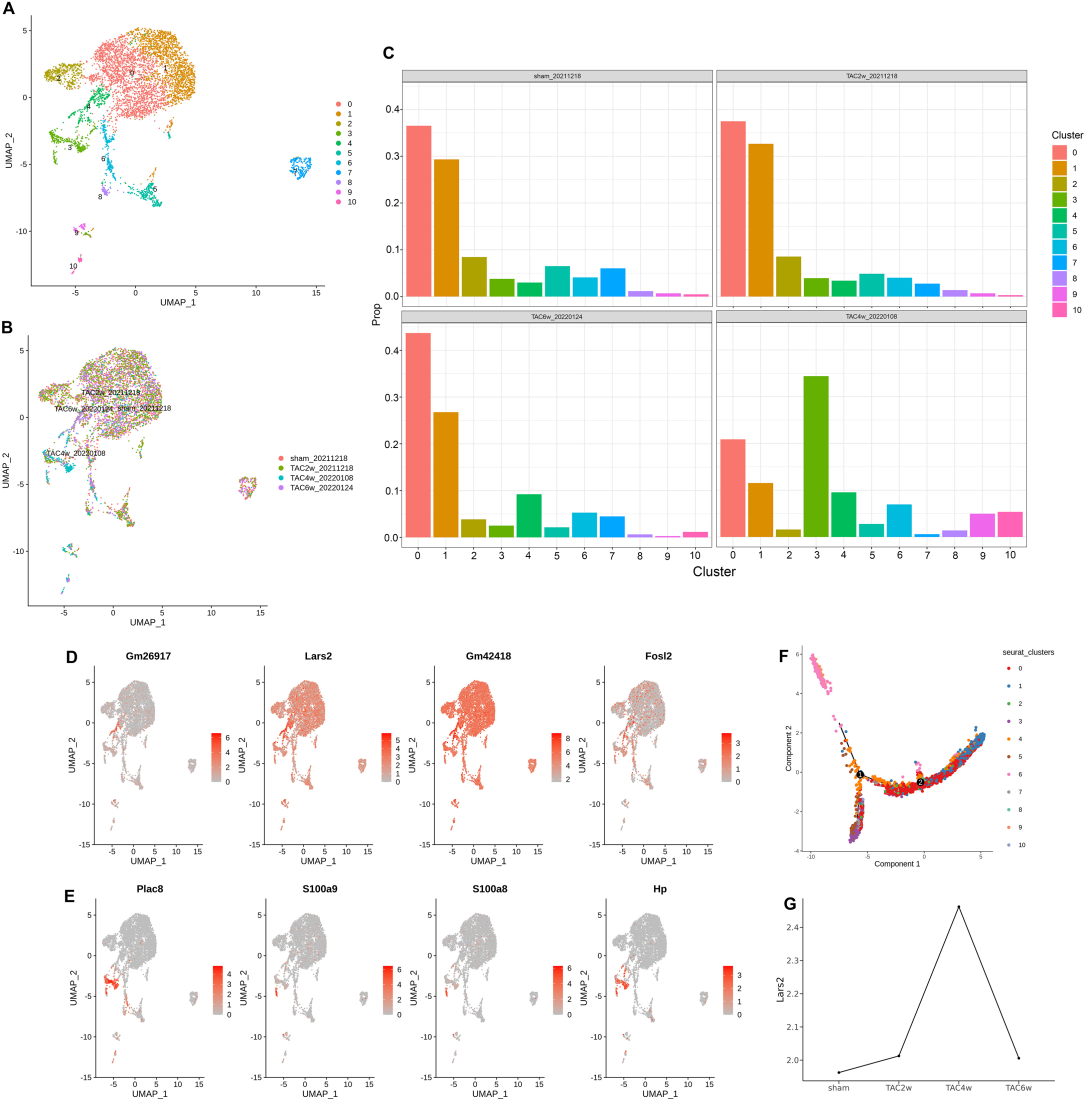
Macrophage cell subsets. **(A)** The UMAP visualization of the cardiac macrophage cell identified sub-sub clusters 0-10. Each point represents a single cell, colored according to the sub-cluster assigned. **(B)** The UMAP visualization of the cardiac macrophage cell sub-sub clusters 0–10 according to the time-point after TAC- operated. **(C)** The proportion of the cardiac macrophage cell sub-sub clusters 0–4 according to the time-point after TAC- operated. **(D)** The UMAP visualization of the typical markers associated with the cardiac macrophage cell sub-sub clusters 4. **(E)** The UMAP visualization of the typical markers associated with the cardiac macrophage cell sub-sub clusters 3. **(F)** Pseudo-time trajectory of the cardiac macrophage cell sub-sub clusters 0-10. **(G)** Gene expression levels of Lars2 in the cardiac macrophage cell according to the time-point after TAC- operated.

Macrophages continue to cluster, obtaining 0–10 cell subsets (**Figure 4A**), and the distribution of these subsets in different stages of TAC (**Figure 4B**). TAC-4w has a relatively unique cell distribution, corresponding to Cluster 3 and 4. Further analysis of the proportions of macrophage subpopulations Cluster 0–10 at different stages of TAC (**Figure 4C**) revealed that Cluster 3 and Cluster 4 showed more obvious changes in TAC-4w, consistent with the results of the cell distribution map. Differentially expressed genes were analyzed for the macrophage subpopulation cells Cluster 0-10, and a series of significantly differentially expressed genes were obtained (**Supplementary Figure S5**), including Lars2, which is significantly represented in the spatial transcriptome. Analysis of Cluster 4 revealed that Lars2 is a significantly differentially expressed gene in Cluster 4 (**Figure 4D**). Gm26917 is a non-coding RNA, Gm4218 is 18s RNA, related to sequence 5, and Fosl2 is tyrosine kinase 2, which is related to cell proliferation. Cluster 3 genes were also analyzed, with C5 and S100a8/a9 showing the most significant changes and being associated with inflammation and coagulation (**Figure 4E**). This study is based on the results of spatial transcriptome analysis, and the changes in Lars2 in macrophage subpopulations are discussed further. A pseudo-time analysis was performed on the macrophage subpopulations Cluster 0–10 to try to understand the point of action of these clusters in the course of the disease. Among them, pseudo-time analysis of macrophage subsets (Clusters 0-10) was conducted to investigate the polarization trajectory of macrophages (M1 pro-inflammatory vs. M2 anti-inflammatory) and determine the role of Lars2-high macrophages in this transition. Cluster 4 cells are the main process of development and evolution and are the main participants in the process of cell development and evolution (**Figure 4F**), and Cluster 4 is involved in both bifurcation points and subsequent processes. Cluster 3 (purple) is involved in the branching stage of cell development and may be a continuation of the later stage of Cluster 4 development. The expression of Lars2 in the heart tissue of mice with different disease courses in the TAC model was analyzed. The expression in individual macrophages was found that TAC-2w expression did not change much compared to sham, TAC-4w expression was the highest, and after 6w it returned to normal or below (**Figure 4G**).

**Figure 5:**
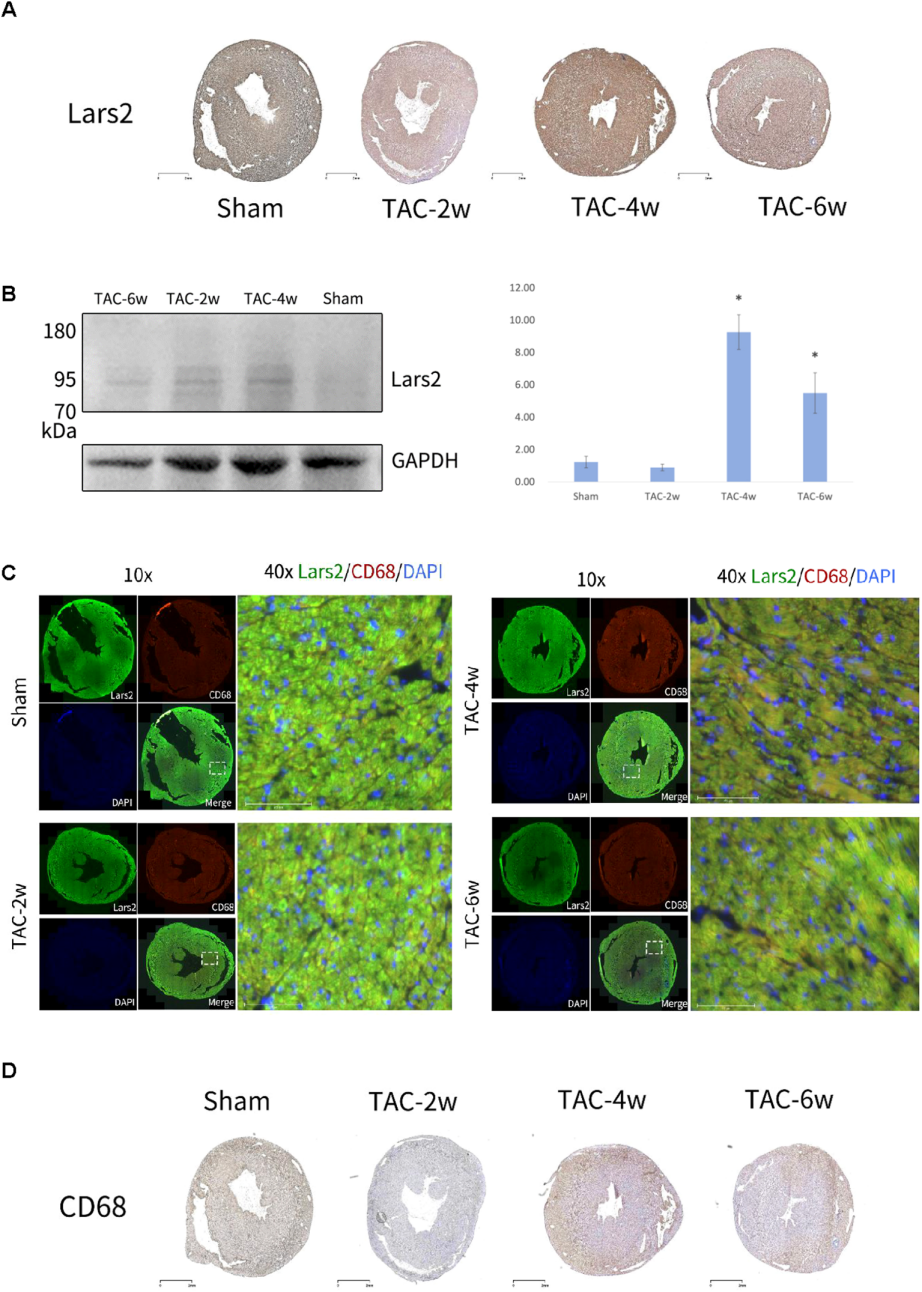
Validation of the expression of Lars2 in TAC- operated mouse heart. **(A)** Immunohistochemistry showed the expression of Lars2 from sham to 6 weeks after the induction of TAC. Results are representative of three different samples. **(B)** Western blot targeting Lars2 from sham to 6 weeks after the induction of TAC. 50 µg of infarcted heart tissue proteins were loaded onto each lane. Quantitative comparison of the Lars2 expression from sham to 6 weeks group (n = 4 for each group). Unpaired two-tailed t-test was used to determine the statistical significance. *P < 0.05, **P < 0.01, ***P < 0.001. **(C)** colocalization assay for Lars2 and the macrophage marker CD68 in the TAC- operated mouse heart from sham to 6 weeks group. Cells were stained with the specific antibodies anti- Lars2 (green) and anti-CD68 (red). Nuclei were counterstained with DAPI (blue). **(D)** Immunohistochemistry showed the expression of CD68 from sham to 6 weeks after the induction of TAC. Results are representative of three different samples.

To validate the expression of Lars2 in TAC-operated mouse hearts, Immunohistochemistry (IHC) staining was performed on heart tissue from TAC mouse models with different disease courses to detect the expression of Lars2 using a Lars2 antibody. IHC showed Lars2 expression was similar between TAC-2w and sham (IOD/area: 0.12 ± 0.02 vs. 0.11 ± 0.01), peaked at TAC-4w (0.35 ± 0.03), and decreased at TAC-6w (0.15 ± 0.02) (**Figure 5A**). As shown in **Figure 5A**. Western blot targeting Lars2 from sham to 6 weeks after the induction of TAC was made (n= 3 per group), and the t test was used to determine statistical significance, p< 0.05. The results showed that the expression of Lars2 in TAC-2w and sham was not significantly different, that TAC-4w was the highest, and that TAC-6w was reduced (**Figure 5B**). Fluorescent immunolabeling of Lars2 in macrophages in heart tissue from TAC mouse models with different disease courses was analyzed (**Figure 5C**). Macrophage marker protein CD68 was used to co-label Lars2 in heart tissue from sham, TAC-2w, TAC-4w, and TAC-6w. Cells were stained with specific antibodies against Lars2 (green) and CD68 (red). The nuclei were counterstained with DAPI (blue). It can also be seen that TAC-2w expression does not change much compared to sham and that TAC-4w has the highest expression, returning to normal levels or below after 6w. IHC staining was performed on heart tissue from TAC mice with different disease courses using anti-CD68 antibodies to detect the expression of the macrophage marker protein CD68. The IHC results showed that CD68 expression was reduced in TAC-2w compared to sham, reached the highest level in TAC-4w, and did not change much in TAC-6w (**Figure 5D**).

### Serum leucine concentration detection

This study constructed a mouse cardiac pressure load model through transverse aortic constriction (TAC), and administered normal feed (NCD) and leucine-deficient feed (LDD) respectively. The serum leucine concentrations at different time points (2 weeks, 4 weeks, 6 weeks) were dynamically detected. As shown in **Supplementary Figure S6**, under NCD feeding conditions, compared with the Sham group, the serum leucine concentration in the TAC-4w group was significantly increased (**P < 0.01), while there were no significant differences in the TAC-2w and TAC-6w groups. This suggests that under normal nutritional conditions, the pressure load induced by TAC can cause a transient increase in serum leucine at 4 weeks; while under LDD feeding conditions (**Supplementary Figure S6**), the serum leucine concentration in the TAC-4w group was significantly lower than that in the Sham group and the TAC-2w group (**P < 0.01), and the TAC-6w group still maintained a low level, indicating that the leucine-deficient diet can alter the time-dependent changes of serum leucine in TAC mice, causing a significant and persistent decrease at 4 weeks. In summary, the serum leucine concentration in TAC mice has a dietary-dependent and time-dependent dynamic change characteristic.

### Effect of leucine-deficient diet on cardiac remodeling in TAC mice

**Figure 6:**
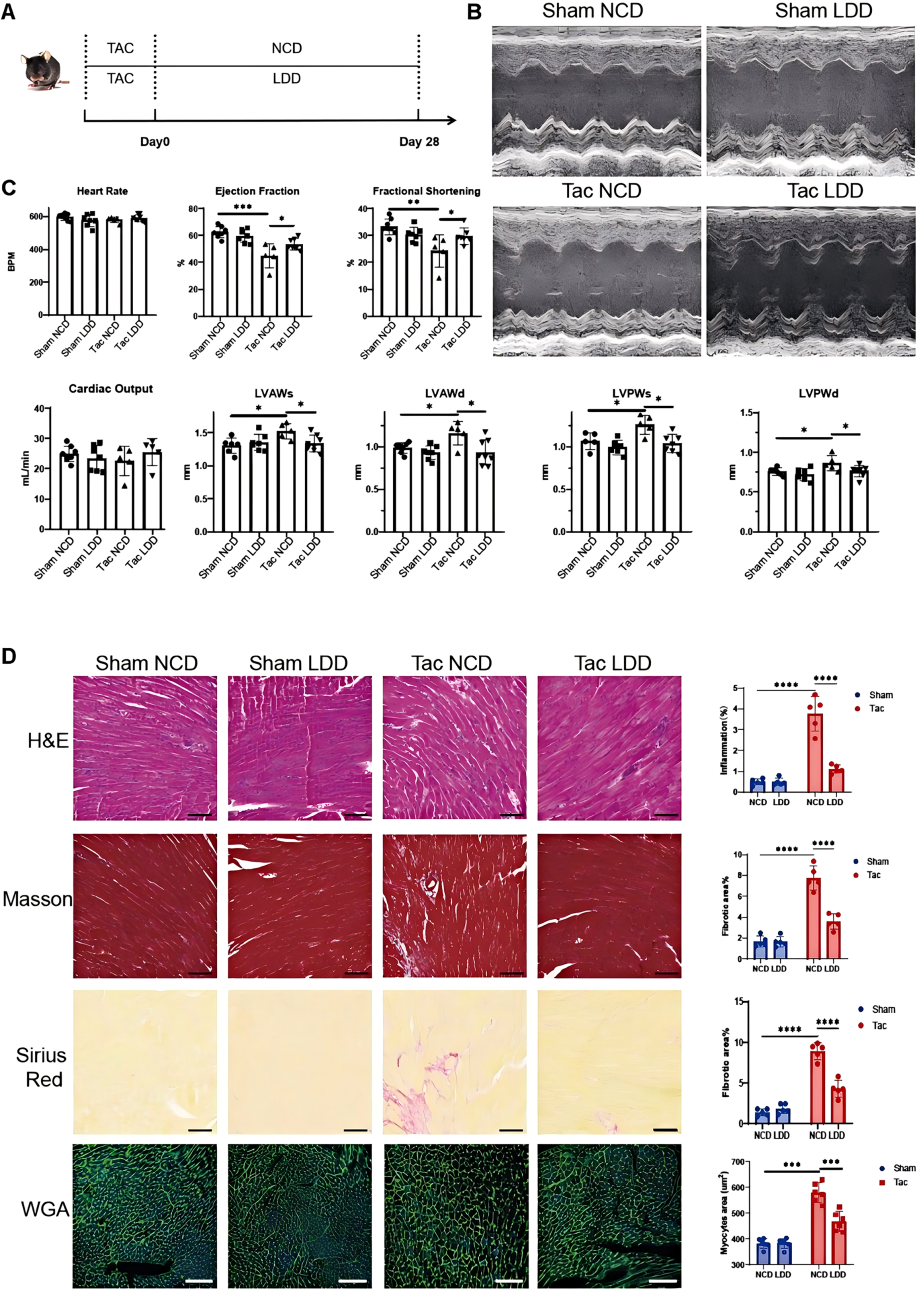
Effect of Leucine dieting on TAC- operated mouse heart. **(A)** Treatment schedule. After TAC-mice modeling (day 0), the mice were randomly assigned to the control group or the leucine-deficient diet (LDD) group. While sham operation mice received the same treatment. **(B)** Echocardiographic results of four groups of mice. **(C)** Quantification of the heart rate (up left), ejection fraction (up middle), fractional shortening (up right), cardiac output (down left) and wall thickness in systolic and diastolic phases (down left). **(D)** Hematoxylin and eosin (H&E) staining (up), masson staining (middle up), Sirius red staining (middle down), wheat germ agglutinin (WGA) staining (down) of heart tissues of four groups. Quantification results of staining were showed on the right. *P < 0.05, **P < 0.01, ***P < 0.001.

Based on the aforementioned research findings, we identified that Lars2 plays a critical role in the pathogenesis of the TAC model. Given that Lars2 is a key molecule in leucine metabolism, we aimed to investigate the impact of a leucine-deficient diet (LDD) on cardiac hypertrophy. Mice were divided into TAC surgery and sham surgery groups. Postoperatively, half of each group received the normal chow diet (NCD), while the other half were subjected to the LDD for four weeks, followed by echocardiographic and pathological evaluations (**Figure 6A**).Echocardiographic results revealed that cardiac ejection function was reduced 4 weeks post-TAC surgery, and this decline was ameliorated by the LDD (**Figures 6B,C**). Furthermore, the LDD also alleviated TAC-induced cardiac hypertrophy, according to the wall thickness of the anterior and posterior walls during systole and diastole (**Figure 6C**). Heart rates across all four groups were comparable during echocardiography, confirming that observed differences in other parameters reflected true structural and functional changes rather than heart rate variability (**Figure 6C**). Subsequently, pathological staining of cardiac tissues included HE staining to assess inflammation, Masson’s trichrome and Sirius red staining to evaluate fibrosis, and WGA staining to quantify cardiomyocyte hypertrophy. Results demonstrated that the LDD significantly ameliorated TAC-induced inflammation and fibrosis while reducing cardiomyocyte hypertrophy (**Figure 6D**).

## Discussion

Pressure overload-induced cardiac remodeling is a pivotal pathological process leading to heart failure, characterized by intricate spatiotemporal crosstalk between parenchymal cells (cardiomyocytes, fibroblasts) and immune cells. However, traditional single-omics approaches—such as single-cell RNA sequencing (scRNA-seq) or spatial transcriptomics (ST) alone—have failed to fully resolve the spatial context of cellular dynamics and molecular regulation, limiting mechanistic insights and therapeutic development (3, 12). In this study, we addressed this gap by integrating ST and scRNA-seq to dissect the spatiotemporal landscape of myocardial remodeling in a transverse aortic constriction (TAC) mouse model, a well-established preclinical model of pressure overload (8, 9). Our findings not only validate and extend prior observations on cell subset dynamics during TAC progression but also identify a novel regulatory axis centered on Lars2 (mitochondrial leucyl-tRNA synthetase) in macrophages—linking leucine metabolism to inflammatory remodeling—and confirm a leucine-deficient diet (LDD) as a targeted therapeutic strategy.

A critical limitation of prior TAC research has been the inability to reconcile cellular heterogeneity (captured by scRNA-seq) with spatial organization—an essential factor for understanding cell-cell communication in the myocardial microenvironment (3). For instance, Alexanian et al. used scRNA-seq to report dramatic shifts in fibroblast states (e.g., activation into myofibroblasts) during TAC progression (20), but this work lacked spatial information, precluding insights into where these state changes occur and how they coordinate with immune cell infiltration. Our integration of ST and scRNA-seq via the SPOTlight algorithm (16) directly addressed this limitation, leveraging the strengths of both technologies: ST retains spatial location, while scRNA-seq provides single-cell resolution (4, 5). Our ST analysis revealed that while cardiac cells are uniformly distributed across myocardial tissue (no focal lesions) in TAC mice, Cluster 1—localized specifically around the left ventricular cavity—exhibited the most pronounced spatiotemporal changes. This cluster’s proportion increased from 8.3% ± 1.5% at TAC-2w to 22.5% ± 1.8% at TAC-6w, and scRNA-seq deconvolution confirmed it is enriched in fibroblasts, macrophages, and T cells. This spatial localization is biologically meaningful: the left ventricle bears the primary burden of pressure overload, and our data position Cluster 1 as a “hotspot” for coordinated parenchymal-immune crosstalk. Complementary scRNA-seq analysis further identified TAC-4w as a critical peak remodeling stage: at this time point, fibroblast and macrophage proportions decreased, while immune cells surged. By TAC-6w, cell composition reverted to TAC-2w levels, reflecting a dynamic “injury-stress-repair” cycle consistent with TAC model pathophysiology (9). Notably, this spatial-temporal coordination would be obscured by either technology alone: scRNA-seq would detect cell proportion changes but not their localization, while ST would identify Cluster 1 but lack single-cell resolution to define its composition. This integration underscores the value of multi-omics approaches in resolving complex tissue remodeling processes (6, 21), a key advance for cardiovascular research.

In this study, the cell population characteristics obtained by scRNA-seq of the heart of the TAC mouse model were used to further analyze the spatial distribution, cell composition, and gene expression characteristics of cardiac tissue cells in the TAC mouse model heart during disease progression using a combination of ST and scRNA-seq techniques. The ST analysis revealed that the distribution of cardiac tissue cells in the TAC mouse model is predominantly a uniform, scattered distribution. As the disease affects the entire myocardium, and specific lesion areas are absent, the cellular changes manifest as a change in the entire region. However, even in such heart tissue devoid of characteristic lesion areas, spatial transcriptome analysis indicates that the cell cluster demonstrating the most significant change with disease progression is Cluster 1. Among the genes that demonstrated significant differential expression in Cluster 1 is Lars2, a gene not previously reported in studies of TAC mouse model heart tissue. To further illuminate the cellular composition of spatial transcriptome Cluster 1, single-cell RNA sequencing (scRNA-seq) was employed. This approach was adopted to mitigate the heterogeneity introduced by the variability in cell numbers, with each spot in the spatial transcriptome containing 1–10 cells. Consequently, single-cell sequencing was performed on the heart tissues of TAC mice with varying disease courses. This analysis yielded 14 distinct single-cell clusters, designated as Cluster 0-14. These single-cell clusters were annotated using existing marker genes, and they were further divided into nine cell subsets, which were essentially divided into two categories: cardiomyocyte subsets related to myocardial composition, endothelial cell subsets, smooth muscle cell subsets, and fibroblast subsets; and macrophage subsets, neutrophil subsets, B cell subsets, T cell subsets, and NK cell subsets related to the microenvironment. The analysis revealed substantial alterations in the proportion of these cell subsets within the heart tissue of different TAC mouse models at TAC-4w, characterized by a decline in the proportion of macrophages and fibroblasts and an increase in the proportion of immune cells and neutrophils. These findings indicate that TAC-4w is the stage at which the most significant changes in the course of the disease occur in the mouse model, with TAC-6w gradually reverting to the state of TAC-2w.

A joint analysis was then performed using Spotlight, integrating the results of the ST analysis and the scRNA-seq results. This analysis yielded the spatial distribution of single-cell subpopulations in the heart tissue of TAC mice at different stages of the disease. Notably, this study observed the distribution of each single cell subpopulation at different stages of the disease, despite the absence of characteristic lesion areas. The myocardial cell subpopulation, endothelial cell subpopulation, and smooth muscle cell subpopulation, which collectively constitute the heart, were found to be relatively stable and densely distributed, with a decrease in TAC-6w. In contrast, the fibroblast subpopulation and macrophage subpopulation were evenly distributed, with a significant increase in TAC-6w. Conversely, the neutrophil subpopulation, B cell subpopulation, T cell subpopulation, and natural killer cell subpopulation exhibited a decline at the onset of the disease course (TAC-2w), followed by a substantial increase at TAC-4w and TAC-6w. These observations underscore the notion that the cells within the microenvironment undergo significant alterations at varying stages of the disease course, thereby serving as a crucial factor influencing its progression. To observe the changes in single-cell subpopulations included in Cluster 1 during different stages of the disease, the analysis found that the cardiomyocyte subpopulation still occupies an important position in Cluster 1 and begins to decrease at TAC-4w. The proportion of endothelial cell subpopulations and smooth muscle cell subpopulations that comprise the myocardium in Cluster 1 remained relatively stable during the disease. The proportion of fibroblast and macrophage subpopulations in Cluster 1 exhibited a gradual increase for the disease. Consistent with previous studies, our scRNA-seq data showed significant changes in fibroblast subsets during TAC progression (22, 23). Specifically, fibroblast proportion decreased at TAC-4w (12.5% ± 1.1% vs. sham 20.3% ± 1.5%) and recovered at TAC-6w, which may reflect the transition from quiescent fibroblasts to activated myofibroblasts at the peak of inflammation (TAC-4w). Unlike previous studies that focused on fibroblast activation alone, our integrated ST-scRNA-seq analysis further revealed that fibroblast dynamics are spatially correlated with macrophage and T cell infiltration (Cluster 1, around the left ventricular cavity), suggesting a coordinated role in myocardial remodeling. Immune cells: The proportion of T cells in Cluster 1 underwent a significant decrease in TAC-2w, a gradual increase in TAC-4w, and a subsequent peak in TAC-6w. Conversely, the trends for neutrophils and B cell subsets exhibited a contrasting pattern, with a peak at TAC-4w followed by a decline at TAC-6w. This trend of change was consistent with the course of the disease, prompting further investigation into the characteristics of this course, specifically whether it involved a decrease in inflammation and an increase in immunity cells accounted for the lowest proportion in Cluster 1 and exhibited minimal change. These results further underscore the influence of microenvironment cells on the course of the TAC mouse model, particularly the pronounced changes in macrophages and T cell subsets.

To further explore the gene information of T cells and macrophage subsets obtained in the spotlight analysis results, a sub-subgroup analysis of T cells and macrophage subsets was performed. The analysis of T cell sub-subgroups found that: Cluster 0–4 cell sub-subgroups were obtained, and Cluster 1 was found to be a relatively unique cell distribution in TAC-4w. Secondly, T cell sub-subgroups Cluster 1 account for a relatively high proportion in TAC at different stages of the disease, and there is a trend of gradual increase. Thirdly, the significantly differentially expressed genes of T cell sub-subgroups Cluster 1 are Lef1, Ccr7, and Sell, which are lymphoid enhancer-binding factor 1, chemokine receptor 7, and cell adhesion molecule, respectively. These genes are closely related to immunity (24–26). Phylogenetic analysis has revealed that these genes are related to the early development and evolution of T cells. Pseudo-time analysis revealed that T cell Cluster 1 (enriched in Lef1/Ccr7/Sell) represents an early activation stage, which gradually accumulates during TAC progression and may mediate adaptive immune responses. For macrophages, Cluster 4 (Lars2-high) is located at the bifurcation point of the pseudo-time trajectory, suggesting it is a key intermediate state driving M1 polarization at TAC-4w. These results clarify the functional significance of T cell and macrophage subset dynamics in disease progression.

The analysis of macrophage subpopulations yielded several notable findings: The presence of 0–10 cell subpopulations was identified, with TAC-4w exhibiting distinct cell subpopulations, particularly Cluster 3 and Cluster 4. Macrophage subsets were classified based on marker gene expression: Cluster 4 (Lars2-high) expresses M1 pro-inflammatory markers (CD68, TNF-α, IL-6) and lacks M2 markers (Arg1, CD206), indicating it is an M1-like pro-inflammatory subpopulation. Cluster 3 (C5/S100a8/a9-high) also exhibits pro-inflammatory characteristics, consistent with previous reports that S100a8/a9 is a key mediator of M1 macrophage activation (27). Cluster 4 macrophages, distinguished by the highest Lars2 expression, were conclusively identified as M1 pro-inflammatory cells: they selectively up-regulated canonical M1 markers (Cd68, Tnf-α), their expansion coincided with the peak systemic pro-inflammatory cytokine surge at TAC-4w, and both infiltration and cytokine release were blunted by LDD. Thus, Lars2 emerges as a hitherto-unrecognized gatekeeper of M1 polarization in pressure-overload cardiomyopathy. A significant shift in the distribution ratio of macrophage subpopulations was observed in the cardiac tissue of TAC models at different stages of the disease, with Cluster 3 and Cluster 4 displaying notable changes in TAC-4w. The differential expression of genes in these subpopulations was further examined, identifying Lars2 and Fosl2 as significantly differentially expressed in Cluster 3 and Cluster 4, respectively. Fosl2, a tyrosine kinase 2, has been implicated in cell proliferation (28), while the significantly differentially expressed genes in Cluster 3 are C5 and S100a8/a9, which are involved in inflammation and blood clotting (29). The proposed time series found that Cluster 4 was involved in the main processes of cell development and evolution, including the bifurcation point and subsequent processes. Cluster 3 was involved in the branching stage of cell development and may be a continuation of the later stage of Cluster 4 development. An analysis of the distribution and expression of Lars2 in the heart tissue of mice with different stages of TAC in macrophages revealed that TAC-2w expression remained relatively stable compared to the sham group, while TAC-4w exhibited the highest expression and returned to normal or below after 6w.

A major gap in prior TAC research has been the understudied role of amino acid metabolism—particularly genes like Lars2—compared to classical inflammatory/fibrotic pathways (e.g., NF-κB, TGF-β) (13). Our integrated analysis identified Lars2 as a significantly differentially expressed gene (DEG) in TAC-induced remodeling, with unique cell-type specificity and temporal dynamics. Lars2 expression followed a stage-specific pattern: no significant difference between TAC-2w and sham, peak expression at TAC-4w, and a decline at TAC-6w (**Figures 5A,B**). This pattern was confirmed by Western blotting (P<0.01) and immunofluorescence co-localization with CD68 (**Figure 5C**), confirming Lars2 upregulation is restricted to macrophages during peak remodeling. Although Lef1, Ccr7, and Sell obtained from T cell subpopulation analysis have not been reported in the TAC mouse model study. Lars2—a novel DEG identified in macrophage scRNA-seq (P<0.001) and ST (P<0.01)—encodes mitochondrial leucyl-tRNA synthetase, which mediates leucine-tRNA binding and mitochondrial ROS homeostasis (18, 30). At TAC-4w, Lars2 upregulation likely promotes pro-inflammatory M1 macrophage polarization, as evidenced by increased CD68^+^ cells (**Figure 5D**) and pro-inflammatory cytokines (e.g., TNF-α, IL-6), linking metabolic dysregulation to myocardial inflammation. Therefore, Lars2 was selected as the object of the verification experiment, WB, and IF experiments were performed on TAC mouse models with different disease courses to study the expression of Lars2. The results of IHC and WB on cardiac tissue from TAC mouse models with different disease courses showed that the expression of Lars2 did not change much between TAC-2w and sham, reached the highest in TAC-4w, and decreased in TAC-6w. Furthermore, the fluorescence immune-location analysis of the co-localization of the macrophage marker protein CD68 and LARS2 in the heart tissue of sham, TAC-2w, TAC-4w, and TAC-6w further substantiates these findings, demonstrating that TAC-2w expression does not exhibit a significant difference compared to sham, TAC-4w expression is maximal, and after 6w, it returns to normal or below. This finding aligns with the results of the spatial analysis, which identified Lars2 as a significantly differentially expressed gene. Concurrently, a trend of decreased Lars2 expression after 6 weeks was observed, with TAC-2w expression approaching that of the sham group. Macrophages exhibited high levels of Lars2 expression. Mechanistically, Lars2 upregulation likely promotes M1 macrophage polarization, a key driver of inflammatory remodeling. TAC-4w mice exhibited increased CD68^+^ M1 cells and pro-inflammatory cytokines, consistent with Lars2-mediated mitochondrial ROS production (30), a known trigger of M1 activation (31). This links metabolic dysregulation (leucine accumulation) to inflammatory remodeling, filling a critical gap in our understanding of TAC pathophysiology, which has historically focused on classical inflammatory pathways (e.g., NF-κB, TGF-β) (13) rather than amino acid metabolism.

This study employed a combination of spatial transcriptomics and single-cell transcriptomics to analyze the cell distribution characteristics, gene composition, and related functions in the heart tissue of the TAC mouse model. Notably, this is the first study to utilize spatial transcriptomics in conjunction with single-cell transcriptomics to characterize myocardial hypertrophy. The results of the study demonstrated that there were alterations in the cell distribution of the heart tissue of the TAC mouse model at different stages of the disease. In addition to cardiomyocytes, the cells in the microenvironment underwent significant changes. The most substantial alterations in the microenvironment were observed in Cluster 1, where the predominant cells were T cells and macrophages. Among the T cells, Lef1, Ccr7, and Sell, and among the macrophages, Lars2, Fosl2, C5, and S100a8/a9 exhibited significant differential expression in these two cell types. The functions of these genes are typical immune functions in T cells and in macrophages, Lars2 is involved in the amino acid synthesis, respiratory chain processes, oxidative stress, and ferroptosis (32–34), Fosl2 is involved in cell proliferation (28), C5 is involved in immune function, and S100a8/a9 is involved in inflammatory processes (29, 32), demonstrating the various related functions of macrophages. Furthermore, IHC, WB, and IF experiments have been conducted to verify that Lars2 changes with the course of the disease. The expression level of TAC-2w is not significantly different from that of sham, and the expression of TAC-4w is the highest. After 6 weeks, a downward trend was observed, which is consistent with the recovery of cell composition changes obtained by single-cell sequencing, which primarily occurred at TAC-4w and TAC-6w. This finding is consistent with the disease process, showing a process of injury, stress, and repair, and gradual stability. The ST combined with the scRNA-seq study identified the Lars2, Lef1, Ccr7, and Sell genes, which have not been previously reported in TAC mouse model studies. The integration of these two novel technologies has facilitated a comprehensive characterization of TAC mouse models in terms of spatial and cellular dimensions. This study has led to the identification of novel research genes and targets, thereby achieving a key objective: the identification of new research avenues within the conventional TAC mouse model using advanced technologies. Our study advances existing knowledge in three key aspects. First, unlike single scRNA-seq or ST studies (35, 36), we integrate both technologies to reveal spatial-temporal coordination between immune cells and fibroblasts. Second, we identify novel DEGs (Lef1, Ccr7, Sell, Lars2) not reported in TAC models, expanding the molecular landscape of myocardial remodeling. Third, we demonstrate that Lars2-mediated M1 polarization links amino acid metabolism to inflammation, a mechanism not previously implicated in pressure-overload cardiomyopathy. These findings bridge gaps between metabolic dysregulation, immune cell function, and spatial organization in cardiac remodeling.

While our findings provide novel insights, this study has several limitations that should be addressed in future work. First, we used a mouse TAC model, which recapitulates key features of pressure-overload cardiomyopathy but may not fully mirror human hypertension or aortic stenosis—particularly in terms of leucine metabolism and immune cell dynamics. Validating Lars2 expression and leucine metabolic profiles in human heart samples (e.g., from patients with aortic stenosis) will be critical for translational relevance. Second, we focused on macrophages as the primary cell type mediating Lars2 function, but leucine metabolism may also regulate other immune cells (e.g., neutrophils) or parenchymal cells (e.g., fibroblasts) in ways not captured here. Third, while LDD shows promise, long-term leucine restriction may have unintended metabolic consequences (e.g., muscle wasting); developing Lars2-specific inhibitors could provide a more precise therapeutic approach. Finally, we did not explore the role of Lars2 in mitochondrial protein synthesis or ROS production directly—future studies using macrophage-specific Lars2 knockout mice will clarify these downstream mechanisms.

This study represents the first integration of ST and scRNA-seq to characterize TAC-induced myocardial remodeling, yielding three key advances: (1) We identified Cluster 1 as a spatial hotspot for coordinated parenchymal-immune cell crosstalk, resolving a critical gap in prior scRNA-seq studies; (2) We discovered Lars2 as a novel regulator linking leucine metabolism to M1 macrophage polarization, expanding the molecular landscape of pressure-overload cardiomyopathy; (3) We validated LDD as a targeted intervention that improves remodeling by downregulating Lars2, providing a translational avenue for treating pressure-overload-induced heart disease. By leveraging multi-omics approaches to uncover spatiotemporal and metabolic-immune mechanisms, our work not only advances basic understanding of cardiac remodeling but also identifies new targets for therapeutic development, core objectives of high-impact cardiovascular research.

## Data Availability

The datasets presented in this study can be found in online repositories. The names of the repository/repositories and accession number(s) can be found below: GSE308859 (GEO).

## Supplementary material

Supplementary Figure 1 The DotPlot shows the most differentially expressed genes in each cluster identified through unsupervised clustering of cardiac cells. Light blue indicates lower expression; dark blue indicates higher expression. Average expression (avg. exp) scale is shown on the right. Known cell type markers strongly and specifically associated with major cell types are shown on the right.

Supplementary Figure 2 Feature plot representing the expression levels of the selected cell-type-specific marker genes.

Supplementary Figure 3 The spatial scatter pie plot with the proportion of each sub-cluster from TAC- operated mouse hearts according to the time-point. The proportions were deconvoluted from the scRNA-seq data using the SPOTlight algorithm. Yellow and red indicate lower and higher proportions.

Supplementary Figure 4 The DotPlot shows the most differentially expressed genes in the cardiac T cell sub-sub clusters 0-4. Blue and red indicate lower and higher expression, respectively.

Supplementary Figure 5 The DotPlot shows the most differentially expressed genes in the cardiac macrophage cell sub-sub clusters 0-10. Blue and red indicate lower and higher expression, respectively.

Supplementary Figure 6 The change in the content of leucine in the serum. **(A)** The concentration of leucine in the serum of TAC mice fed with the normal chow diet (NCD). **(B)** The concentration of leucine in the serum of TAC mice fed with the leucine-deficient diet (LDD).
