# Supplementary figures and images for: Integrated spatial transcriptomics and single-cell RNA sequencing reveal Lars2-mediated spatiotemporal dynamics of myocardial remodeling in a mouse model of transverse aortic constriction
Source: Front Immunol. 2026 Mar 4;17:1701776. doi: 10.3389/fimmu.2026.1701776 (PMC12996057; doi:10.3389/fimmu.2026.1701776)

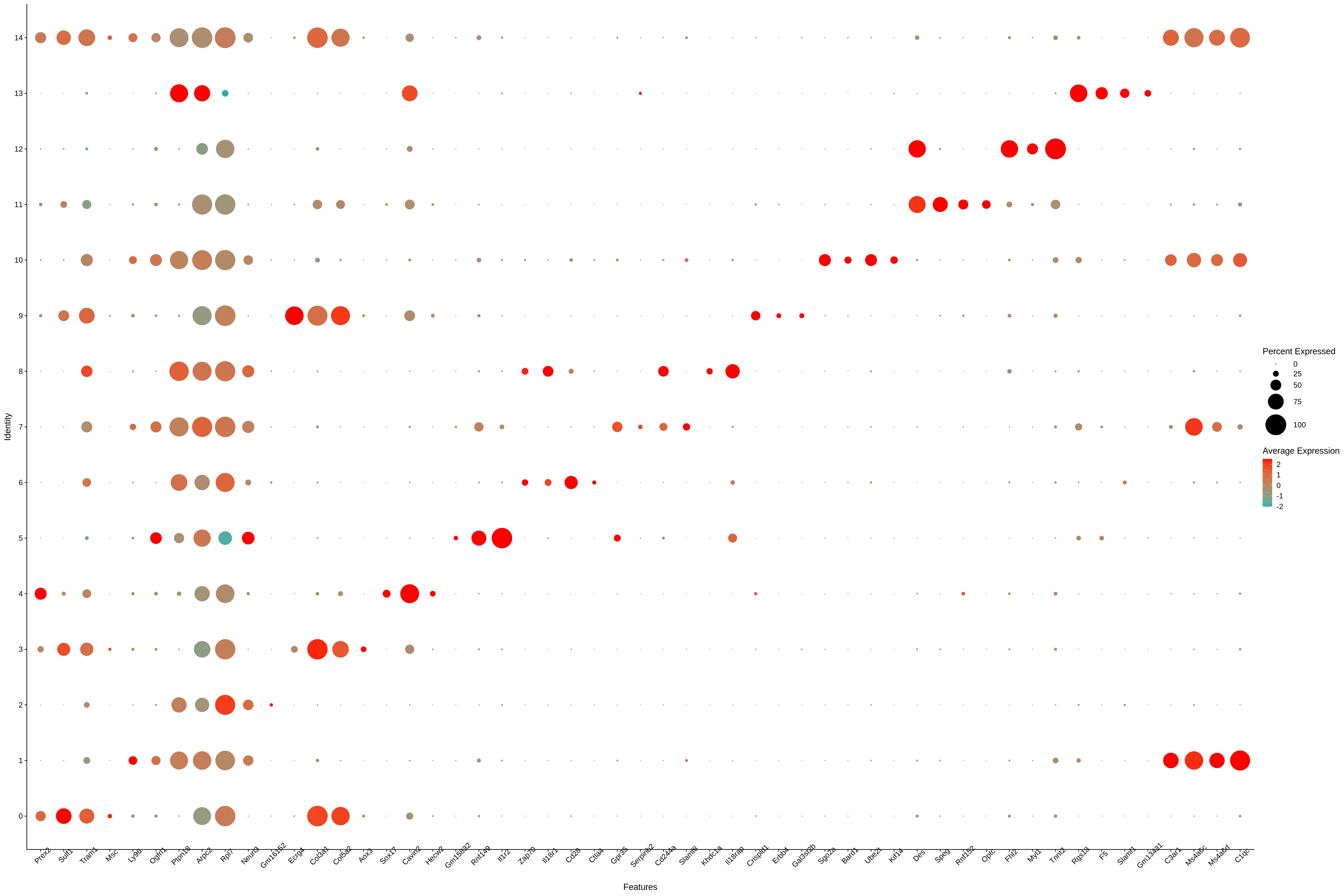

Supplement: Supplementary Figure 1 — The DotPlot shows the most differentially expressed genes in each cluster identified through unsupervised clustering of cardiac cells. Light blue indicates lower expression; dark blue indicates higher expression. Average expression (avg. exp) scale is shown on the right. Known cell type markers strongly and specifically associated with major cell types are shown on the right. [file Image1.jpeg]

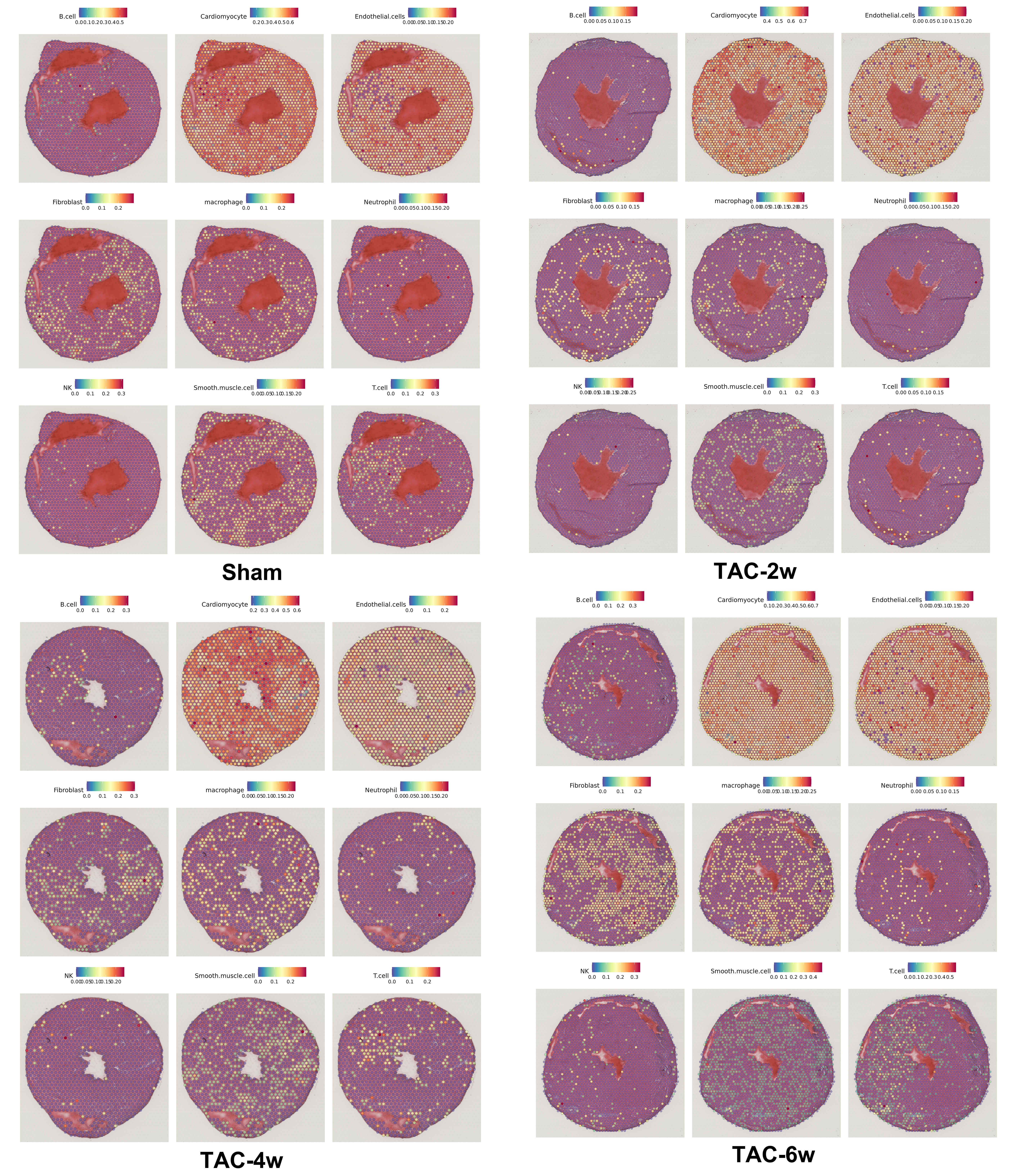

Supplement: Supplementary Figure 3 — The spatial scatter pie plot with the proportion of each sub-cluster from TAC- operated mouse hearts according to the time-point. The proportions were deconvoluted from the scRNA-seq data using the SPOTlight algorithm. Yellow and red indicate lower and higher proportions. [file Image3.jpeg]

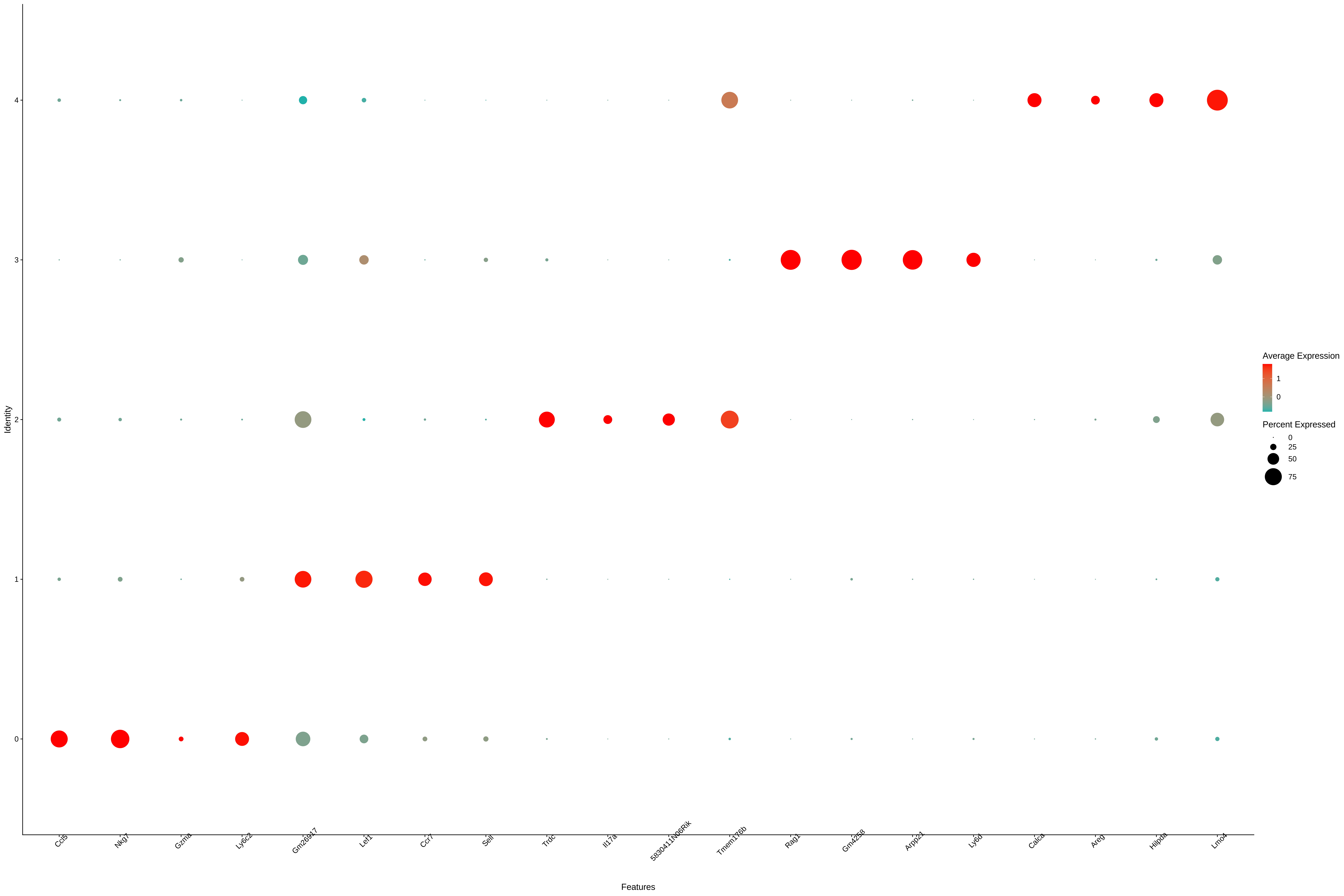

Supplement: Supplementary Figure 4 — The DotPlot shows the most differentially expressed genes in the cardiac T cell sub-sub clusters 0-4. Blue and red indicate lower and higher expression, respectively. [file Image4.jpeg]

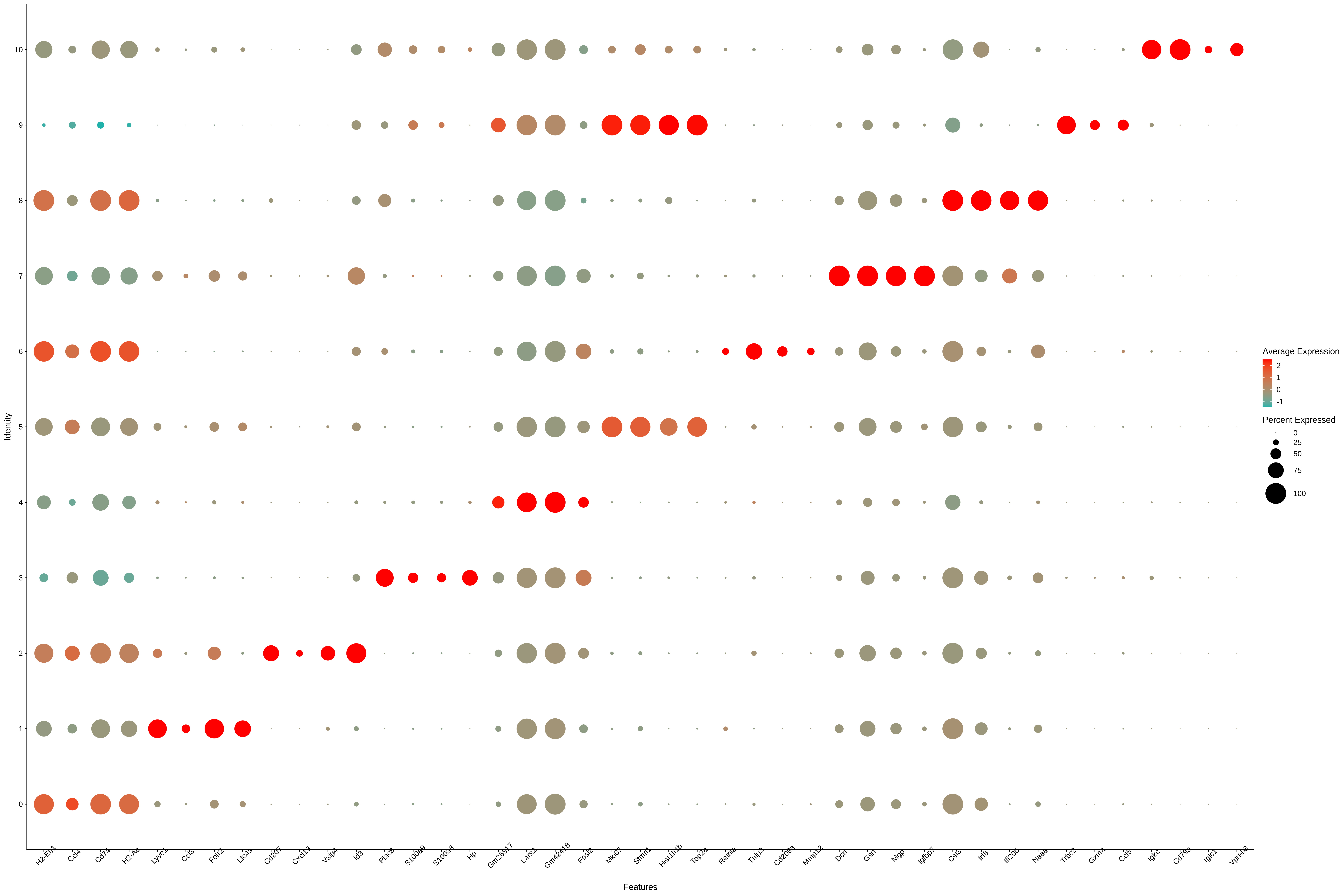

Supplement: Supplementary Figure 5 — The DotPlot shows the most differentially expressed genes in the cardiac macrophage cell sub-sub clusters 0-10. Blue and red indicate lower and higher expression, respectively. [file Image5.jpeg]

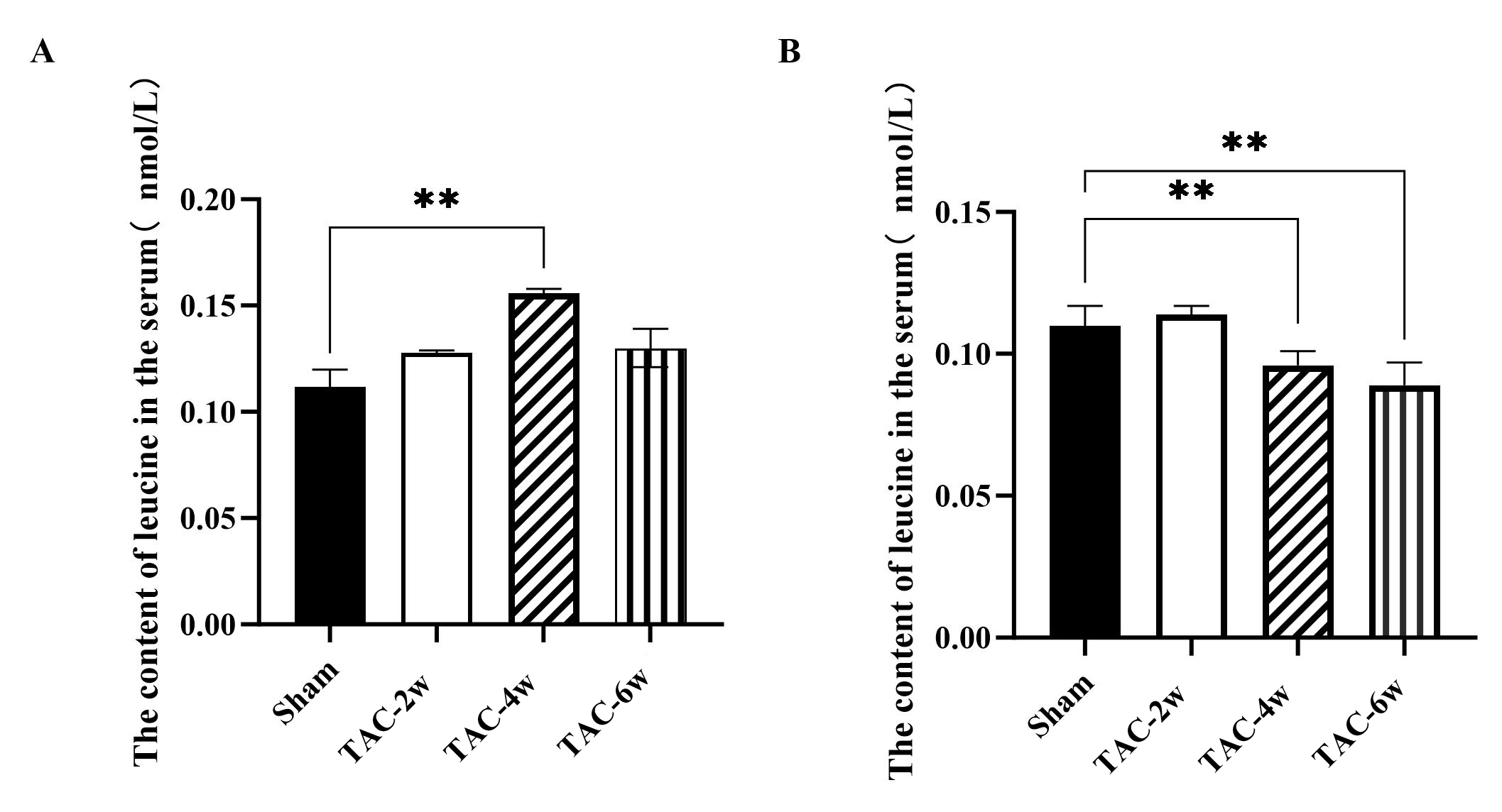

Supplement: Supplementary Figure 6 — The change in the content of leucine in the serum. (A) The concentration of leucine in the serum of TAC mice fed with the normal chow diet (NCD). (B) The concentration of leucine in the serum of TAC mice fed with the leucine-deficient diet (LDD). [file Image6.jpeg]
